# Intracranial Aneurysms Induced by *RUNX1* Through Regulation of *NFKB1* in Patients With Hypertension-An Integrated Analysis Based on Multiple Datasets and Algorithms

**DOI:** 10.3389/fneur.2022.877801

**Published:** 2022-05-17

**Authors:** Yang Li, Zhen Zhang, Donghua Liu

**Affiliations:** ^1^Department of Neurosurgery, The First People's Hospital of Yinchuan, Yinchuan, China; ^2^Department of Neurosurgery, The Affiliated Yantai Yuhuangding Hospital of Qingdao University, Yantai, China; ^3^Department of Neurosurgery, The Second People's Hospital of Yinchuan, Yinchuan, China

**Keywords:** intracranial aneurysm (IA), hypertension, monocytes/macrophages, Runx1, NFKB1

## Abstract

**Objective:**

The purpose of this study was to identify potential therapeutic targets by examining the hub genes contributing to progression of intracranial aneurysm (IA) in patients with hypertension.

**Methods:**

The bulk RNA sequencing (RNA-seq) datasets of hypertension and IA were obtained from the Gene Expression Omnibus (www.ncbi.nlm.nih.gov/geo) database. These data were then used to calculate disease-related differentially expressed genes (DEGs) at the individual level. An scRNA-seq dataset of patients with abdominal aortic aneurysms (AAA) was used to analyze monocyte/macrophage-related DEGs. On the basis of the DEG data related to monocytes and macrophages, a TF-genes network has been developed. Hub genes and core sub-networks have also been identified. Furthermore, the key genes have been validated in an external cohort.

**Results:**

From combined monocyte and macrophage-derived DEGs from abdominal aortic aneurysms, five hub DEGs were detected, including *IFI30, SERPINE1, HMOX1, IL24*, and *RUNX1*. A total of 57 genes were found in the IA bulk RNA-seq dataset. A support vector machine-recursive feature elimination algorithm (SVM-RFE) was applied to further screen the seven genes (*RPS4Y1, DDX3Y, RUNX1, CLEC10A, PLAC8, SLA, and LILRB3*). *RUNX1* was the hub gene that regulated *NFKB1* in the monocyte/macrophage-related network. And *RUNX1* is implicated in IA progression by regulating hematopoietic stem cell differentiation and abnormal platelet production, according to gene set enrichment analysis.

**Conclusion:**

Among patients with hypertension, *RUNX1* in monocytes and macrophages was associated with a higher risk of IA through its regulation of *NFKB1*.

## Introduction

Intracranial aneurysm (IA) is a cerebrovascular disease caused by the limited dilation of cerebral arteries (1). Its incidence is approximately 3.2%, and it is higher in women than in men (2–4). IA ruptures and bleeding are associated with high morbidity rates and mortality rates (5). Subarachnoid hemorrhage (SAH) results from an IA rupture or bleeding, and accounts for 70–85% of all spontaneous SAHs (6). In addition, IA can induce intracerebral or subdural hematoma (7, 8). Hypertension is a common risk factor for IA as well as an independent risk factor for IA rupture (9–11). IA poses a significant risk to patients with hypertension. Therefore, IA progression in this patient group should be prevented.

The role played by monocytes/macrophages in the pathogenesis of IA has been demonstrated in recent studies. Development of IA involves complex pathophysiological processes, such as endothelial inflammatory responses triggered by altered hemodynamics and genetic factors (12, 13). Changes in cerebral artery hemodynamics can trigger a prolonged and excessive inflammatory response in the vessel wall, leading to the development, growth, and rupture of IA (13). Cell death in the vessel wall and destruction of the extracellular matrix can occur as a result of this chronic inflammation caused by the recruitment of monocytes/macrophages (14). During inflammation, monocytes can infiltrate the vessels and become macrophages, which modulates the immune response (15). Macrophages of the M1 type can release cytokines to increase the inflammatory response and recruit further macrophages (16). It is also possible for M1-type macrophages to remodel the blood vessels in addition to releasing cytokines (17). By inhibiting macrophage recruitment and accumulation in the vessel wall of individuals with IA, the incidence and size of IA in animal models will be significantly reduced (18). Monocytes and macrophages play an important role in IA pathogenesis, but the mechanism is not known.

Cells are the basic unit of life, which can communicate via two distinct pathways (paracrine and autocrine) to regulate metabolism, differentiation, and other biological functions (19). Monocytes and macrophages in a hypertensive microenvironment may cause IA through scRNA-seq analysis. The rapid development of single-cell sequence technologies has allowed previous studies to collect gene expression data at the single-cell level in recent years (20–24). The analysis of single cells can identify the mechanisms of cellular interactions and is essential for establishing the molecular regulation of diseases at the microscopic level (25–28). Single-cell RNA sequencing analysis revealed that megakaryocytes and a few monocyte subpopulations may significantly elevate cytokine levels in patients with severe coronavirus disease 2019 (29). We hypothesized that single-cell analysis could provide insight into possible mechanisms underlying the development of hypertension-induced IA.

This study examined hub genes that contribute to IA progression among patients with hypertension and identified potential therapeutic targets for preventing and reducing the risk of IA, thereby lowering the risk of neurovascular diseases including cerebral hemorrhage.

## Methods

### Data Acquisition

First, the Gene Expression Omnibus (GEO) database was searched for IA-related bulk and single-cell RNA-seq datasets. To investigate the possible role of hypertension in IA development, the keyword “Hypertension” was retrieved from the GEO dataset to obtain HT-related datasets. The filtering criteria for the datasets were as follows: First, bulk RNA-seq datasets were derived from human samples, and each dataset contained at least 10 samples. Second, single-cell RNA-seq datasets were assigned as the control design. Transcriptomic data from the peripheral blood mononuclear cells (PBMCs) of 11 patients with hypertension and 10 healthy controls (HCs) were collected from the GSE75360 dataset to analyze hypertension-related differentially expressed genes (DEGs) according to the set conditions (30). The mRNA transcriptomic data from 44 IA samples and 16 HC samples of the intracranial cortical artery from the GSE122897 dataset were used to analyze IA-related DEGs. The GSE13353 dataset contains 11 ruptured and 8 unruptured intracranial aneurysm samples, and this dataset was used as an external validation cohort in this study (31). To assess the transcription of AI-related genes at the single-cell level, we included a single-cell RNA-seq dataset (Gene Expression Omnibus accession no. GSE166676) with four cases of abdominal aortic aneurysm (AAA) and two cases of non-aneurysmal control (NAC) among patients with atherosclerotic occlusive disease after a cautious examination (32). The single-cell RNA sequencing (scRNA-seq) dataset contains transcriptomic data from 14,088 cells. All analyses and plots were created using R (version 4.0.2). Differences between groups were assessed using the Wilcoxon rank-sum test.

### Quality Control and Data Merging of ScRNA-Seq Data

First, the R package Seurat (version 4.0) was used for the quality control of scRNA-seq data from AAA and NAC samples (33). The metrics used for quality control included the number of gene signatures detected in each cell, total RNA count, and proportion of mitochondrial and hemoglobin genes expressed. High-quality data from the scRNA-seq dataset were screened by removing cells containing <200 or > 2,500 genes as well as cells with > 10% of mitochondrial genes. Finally, we obtained 9,796 cells for subsequent analysis. The “SelectIntegrationFeatures” function was then used to identify the top 2,000 highly variable genes shared among the six samples. Further, the “FindIntegrationAnchors” function was then used to find the anchors from the highly variable genes. Finally, by applying the “IntegrateData” function, the scRNA-seq data from the six samples were combined for subsequent dimension reduction and clustering analysis.

### Dimensionality Reduction, Clustering, and Annotation of scRNA-seq Data

The uniform manifold approximation and projection (UMAP) algorithm was used to analyze and visualize cell clustering (34). The top 30 principal components (PCs) were selected to further perform UMAP and clustering analysis. We first used SingleR (version 1.4) to predict the cell types of individual cell clusters (35). The Database of Immune Cell Expression (DICE) and Monaco Immune Cell Data were selected as the reference datasets to predict the types of immune cells in the IA single-cell dataset. Then, the cell type predictions were manually validated against the marker molecules of immune cells from the CellMarker (http://biocc.hrbmu.edu.cn/CellMarker/index.jsp) website to complete the final cell annotation.

### Extraction and Differential Analysis of Target Cell Populations

According to a literature review, monocytes/macrophages play an essential role in IA progression (36). Therefore, we further included monocytes/macrophages for subsequent analysis. First, the RNA transcripts of all genes in the monocytes/macrophages between the AAA and NAC groups were subjected to differential expression analysis. Two approaches were used to identify DEGs between the two groups. The first approach applied the “FindMarker” function to identify DEGs between the two cell populations. The other approach used DESeq2 to the constructed pseudo-bulk RNA data after establishing the pseudo-bulk RNA-seq data for differential expression analysis. DEGs identified with these two approaches were combined as monocyte/macrophage-related DEGs.

### Differential Analysis of Bulk RNA and Identification of Shared DEGs

Based on the type of bulk RNA-seq data, the limma or edgeR package was used for differential expression analysis. We applied the limma package for the differential analysis of FPKM-type RNA data and obtained IA-related DEGs (37). The R package “edgeR” was used to identify hypertension-related DEGs and to perform the differential analysis of count-type RNA data (38). DEGs were screened with a threshold of *P* < 0.05. Subsequently, we performed intersection analysis of IA-related DEGs, hypertension-related DEGs, and monocyte/macrophage-related DEGs to obtain shared DEGs, which were considered as genes of interest (GOIs).

### Support Vector Machines–Recursive Feature (SVM-RFE) Elimination Model Construction and Variable Selection

Intersection analysis of IA-related DEGs and monocyte/macrophage-related DEGs revealed 57 shared DEGs. Then, using the bulk RNA-seq data for IA, we incorporated these 57 genes into the Support Vector Machines–Recursive Feature Elimination (SVM–RFE) model to predict the incidence of IA. This model can be applied to select the optimal number of variables that must be included in the model and can yield the most relevant variables for classification prediction (39).

### TF-Gene Network Construction and Functional Pathway Enrichment Analysis

To explore the significant TF regulatory network in monocytes/macrophages, we applied the TRRUST (version 2.0) database to predict the TF–gene pairs corresponding to monocyte/macrophage-related DEGs (40). Cytoscape (version 3.9) was used to visualize this TF–gene network, and the Molecular Complex Detection plug-in was further used to identify the core sub-networks in this network (degree cutoff = 2, node score cutoff = 0.2, and max depth = 100).

To explore the potential pathways involved in DEGs, we used the clusterProfiler R package for Kyoto Encyclopedia of Genes and Genomes and Gene Ontology (GO) analysis as previous research (41, 42). Gene Set Enrichment Analysis (GSEA) was applied to analyze the functional pathways of enrichment of core genes at the individual and cellular levels. The “c2.cp.v7.2.symbols.gmt [Curated]” gene set downloaded from MSigDB Collections (https://www.gsea-msigdb.org/gsea/msigdb/) was used as the reference gene set. The filtering threshold for differences in functional pathways was set at *P* < 0.05 (43).

## Results

### Quality Control and Annotation Results for scRNA-seq Data

**Figure 1:**
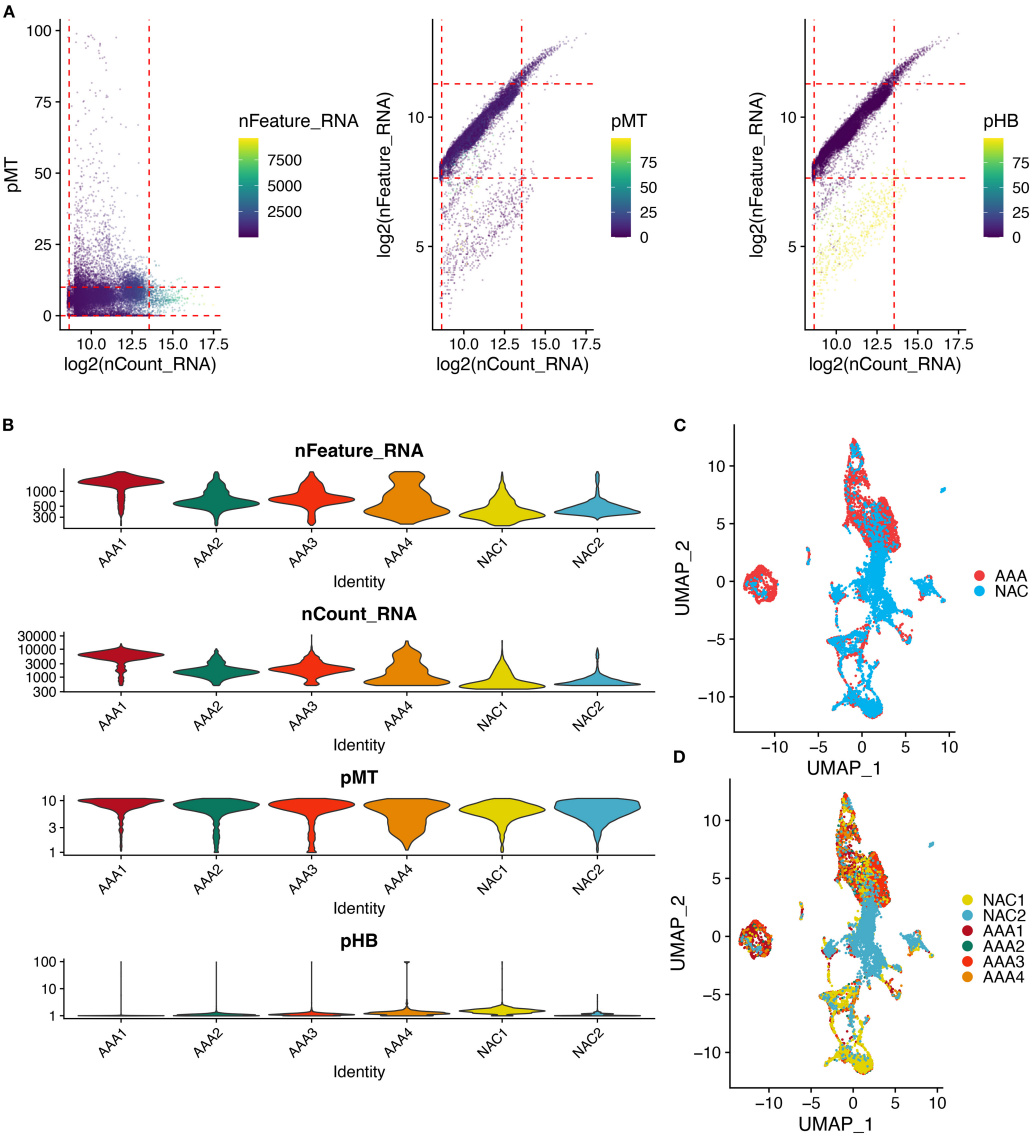
Quality control and cell clustering of single cell data. **(A)** Scatter plot of quality control metrics for scRNA-seq data. The red dashed line represents the threshold used to filter out high quality transcriptomic data. pMT: percent of mitochondrial counts; pHB: percent of hemoglobin RNA counts. **(B)** Violin plots showing the distribution of cell characteristics after quality control. **(C,D)**. UMAPs show the results of cell clustering based on the top 30 principal components, color coded by **(C)** tissue type, or **(D)** patient.

**Figure 2:**
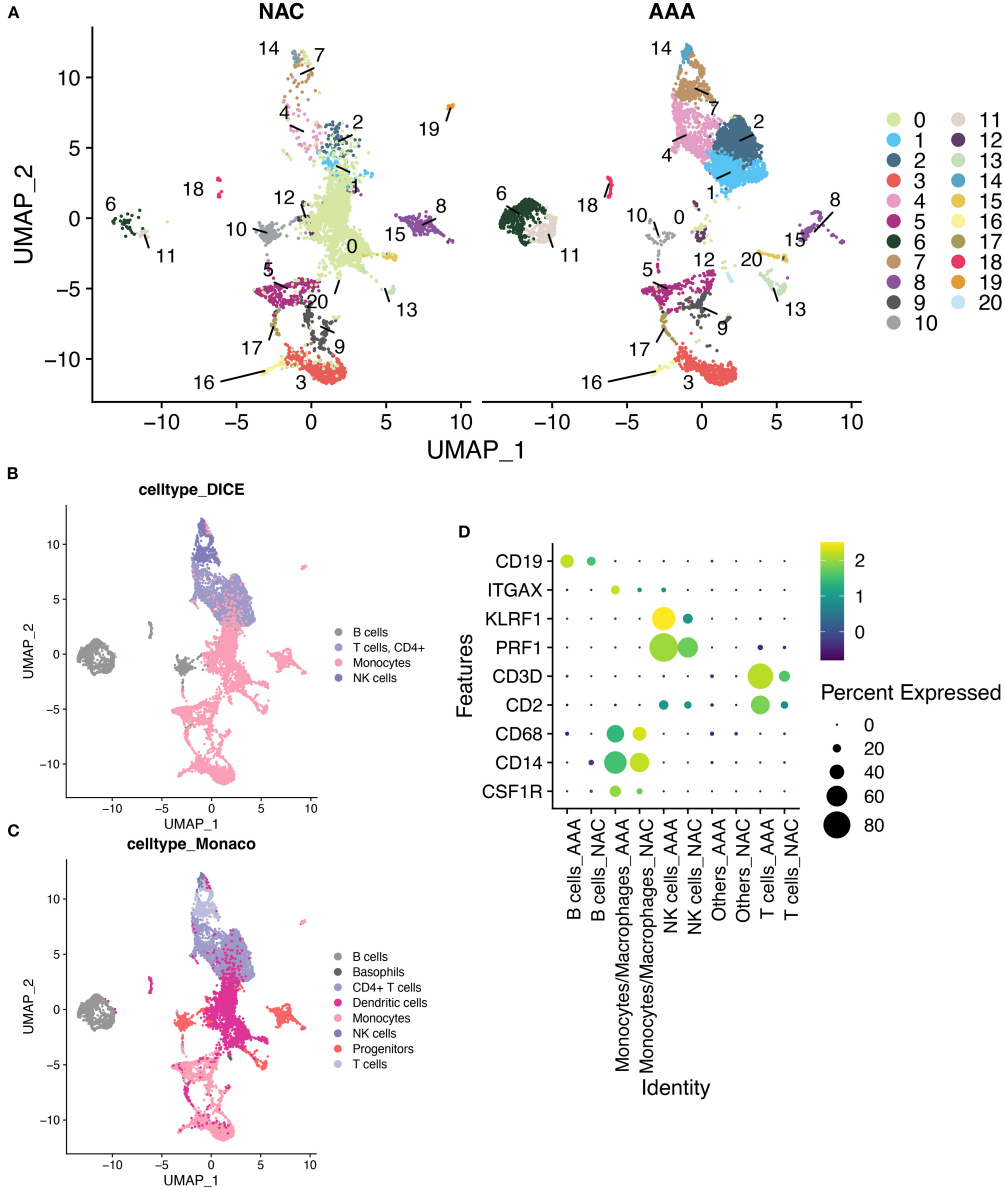
SingleR annotation of cell clusters. **(A)** The UMAPs show a distribution of 21 cell clusters in the AAA and NAC groups, respectively. **(B)**. Application of cell annotation to the DICE reference dataset.DICE: Database of Immune Cell Expression (/eQTLs/Epigenomics). **(C)** Cell annotation using Monaco Immune Cell Data. **(D)** Heat map showing good concordance in the expression of cellular marker genes between AAA and NAC groups.

**Figure 3:**
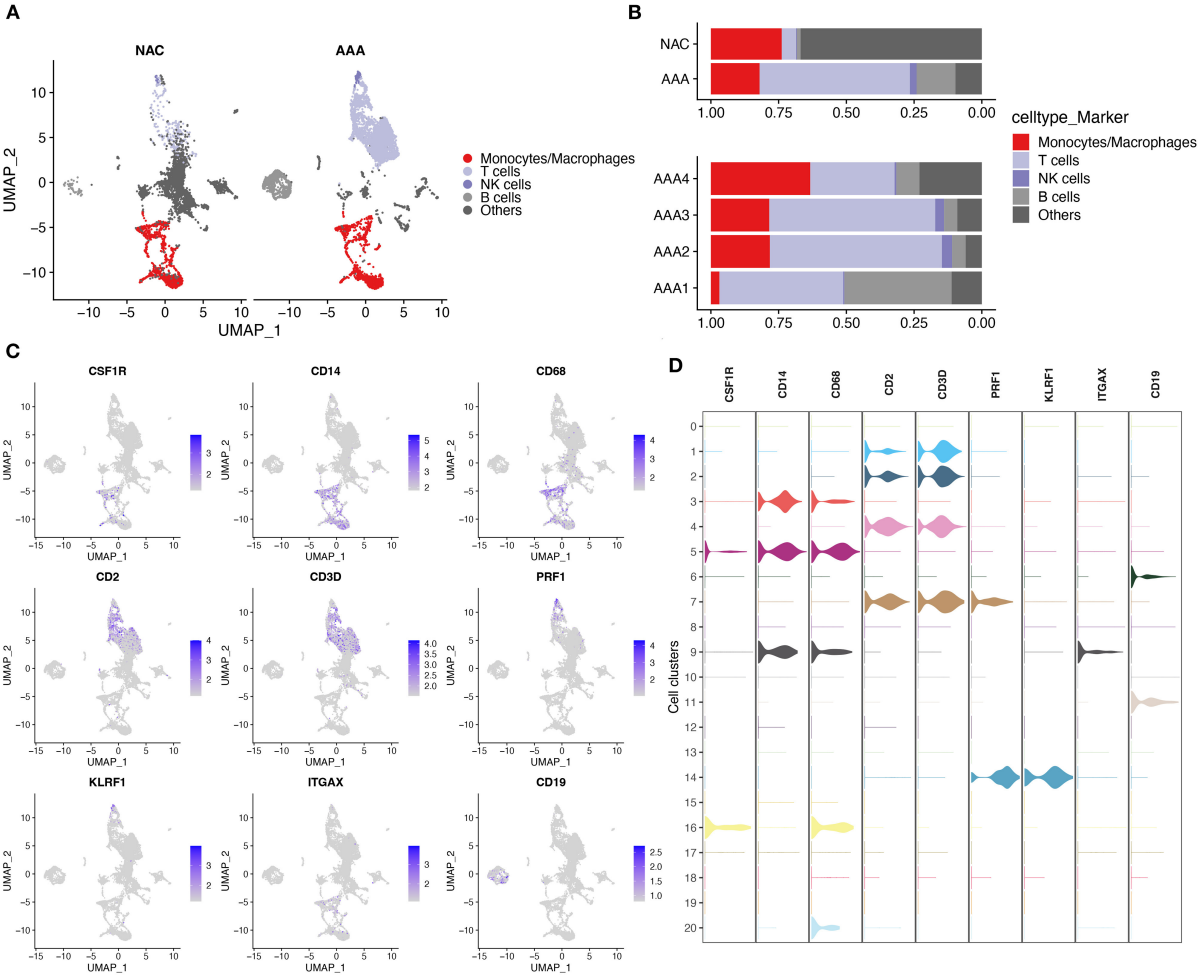
Cell annotation in conjunction with CellMarker. **(A)** UMAPs show the results of applying the marker genes in CellMaker to annotate cells. **(B)** The bars depict the percentage of each cell type in each tissue type and patient. **(C)** UMAPs show the expression of cellular marker genes in cells. **(D)** Violin diagram depicting cell marker gene expression in each cell cluster.

First, we performed quality control on the scRNA-seq data of arterial tissue. The Figure 1A shows the distribution of total NRA counts, the number of gene signatures, and the proportion of mitochondrial gene expression and hemoglobin-related gene expression in cells before filtration. Following the pre-defined filtering conditions, 9,796 cells were included in the subsequent analysis (Figure 1B). UMAP showed that each cell cluster overlapped between the two groups and between the six samples. Therefore, data integration eliminated the batch effect (Figures 1C,D). Data were reduced in dimension and clustered into 21 cell clusters (Figure 2A). Figures 2B,C show four and eight cell clusters annotated using the DICE and Monaco reference datasets, respectively. Among them, monocytes had a relatively large overlap between the two annotation methods. Subsequently, we applied cellular annotation with nine cellular marker genes from CellMarker, which were consistently expressed between the two groups (Figure 2D). Figures 3A,B illustrate the expression of these nine cell markers within each cluster of cells. Among them, CSF1R was used to annotate monocytes/macrophages, CD14 to annotate monocytes, CD68 to annotate macrophages, CD3D and CD2 to annotate T cells, PRF1, and KLRF1 to annotate NK cells, ITGAX to annotate dendritic cells, and CD19 to annotate B cells. Finally, four cell types were annotated by combining the cell annotation results of SingleR and CellMarker, including T cells, B cells, NK cells, and monocytes/macrophages (Figure 3C). A significant difference in the percentage of T cells was observed between the AAA and NAC groups (*p* 0.05) (Figure 3D). In addition, the proportion of monocytes/macrophages differed significantly between samples in the AAA group. Thus, monocytes/macrophages might be involved in the different stages of IA progression.

### Results of Differential and Intersection Analyses

**Figure 4:**
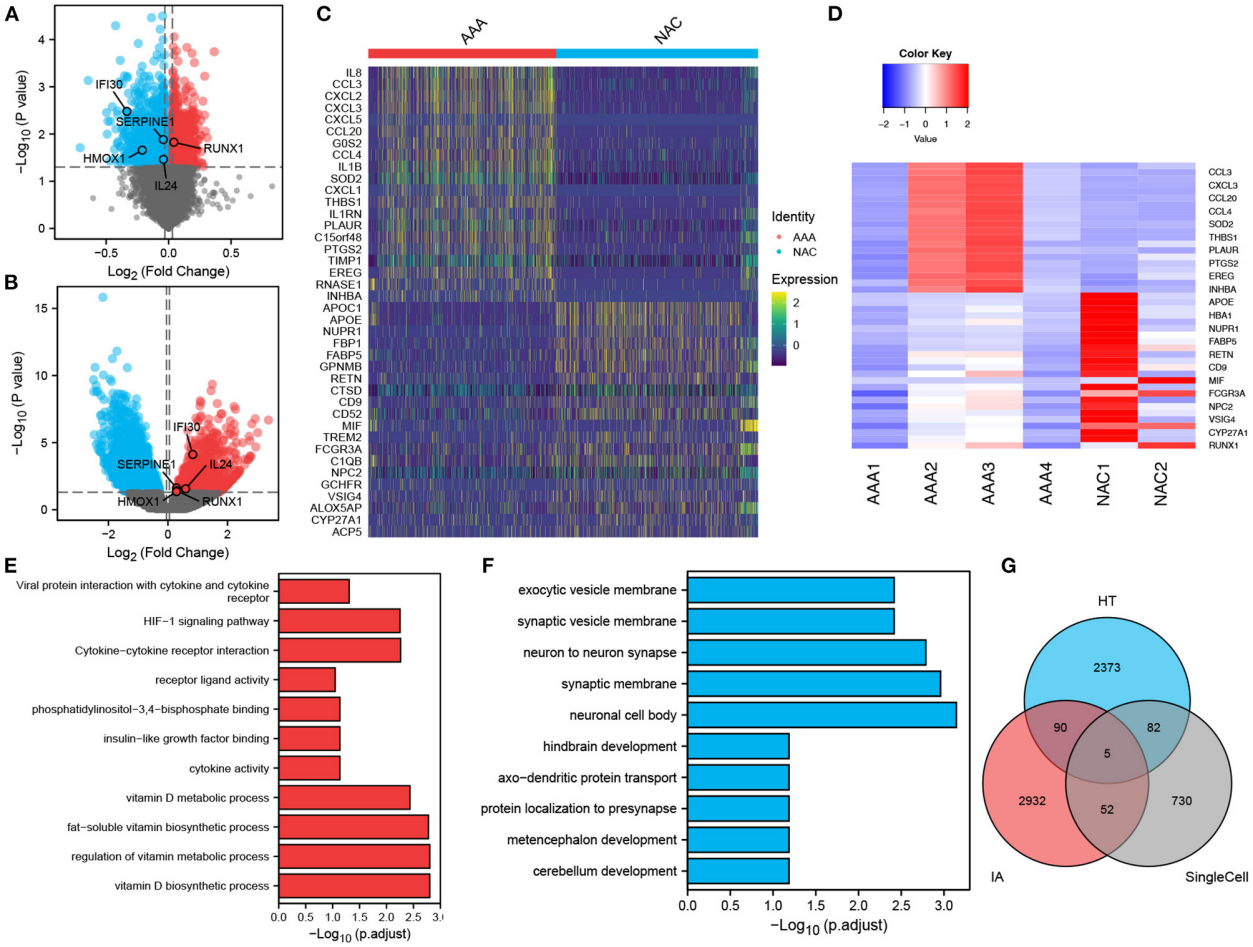
Differentially expressed genes and intersection analysis. **(A,B)** Volcano plots showing the results of tests for differentially expressed genes in **(A)** HT, and **(B)** IA, respectively, and the differences in expression of GOIs are marked in the plots. **(C,D)**. Differentially expressed genes in monocytes/macrophages obtained by applying **(C)** FindMarker, or **(D)** DEGs. **(E,F)** Differential gene-enriched (E) up-regulation pathway and (F) down-regulation pathway shared in HT and IA. **(G)** Intersection analysis of HT-associated DEGs, IA-associated DEGs and monocytes/macrophages-associated DEGs revealed five GOIs.

In total, 2,102 monocytes/macrophages were extracted and used for differential analysis between the AAA and NAC groups. Further, DESeq2 and FindMarker identified 419 and 539 DEGs, respectively, with a total of 869 genes after removal of intersections. Then, 2,550 and 3,079 DEGs were identified from the bulk RNA-seq data of HT and IA, respectively. These genes of interest (GOIs) were annotated in the volcano map (Figures 4A,B). The top 20 upregulated and downregulated DEGs in AAA identified by FindMarker and DESeq2, respectively, are shown in Figures 4C,D. To further explore the functional pathways that are jointly dysregulated in hypertension and IA, GO function enrichment analysis of the 95 DEGs common to HT and IA was performed. Figures 4E,F show the pathways that are jointly upregulated and downregulated in hypertension and IA, respectively. Finally, cross-tabulation analysis revealed that five GOIs, including *IFI30, SERPINE1, HMOX1, IL24*, and *RUNX1*, were found to be associated with hypertension, IA, and monocytes/macrophages (Figure 4G). And the differential expression of *IFI30, SERPINE1, HMOX1, IL24*, and *RUNX1* were shown in the volcano plot (Figures 4A,B).

### SVM–RFE Analysis of the Expression Distribution of Hub Genes in the Bulk RNA of IA

**Figure 5:**
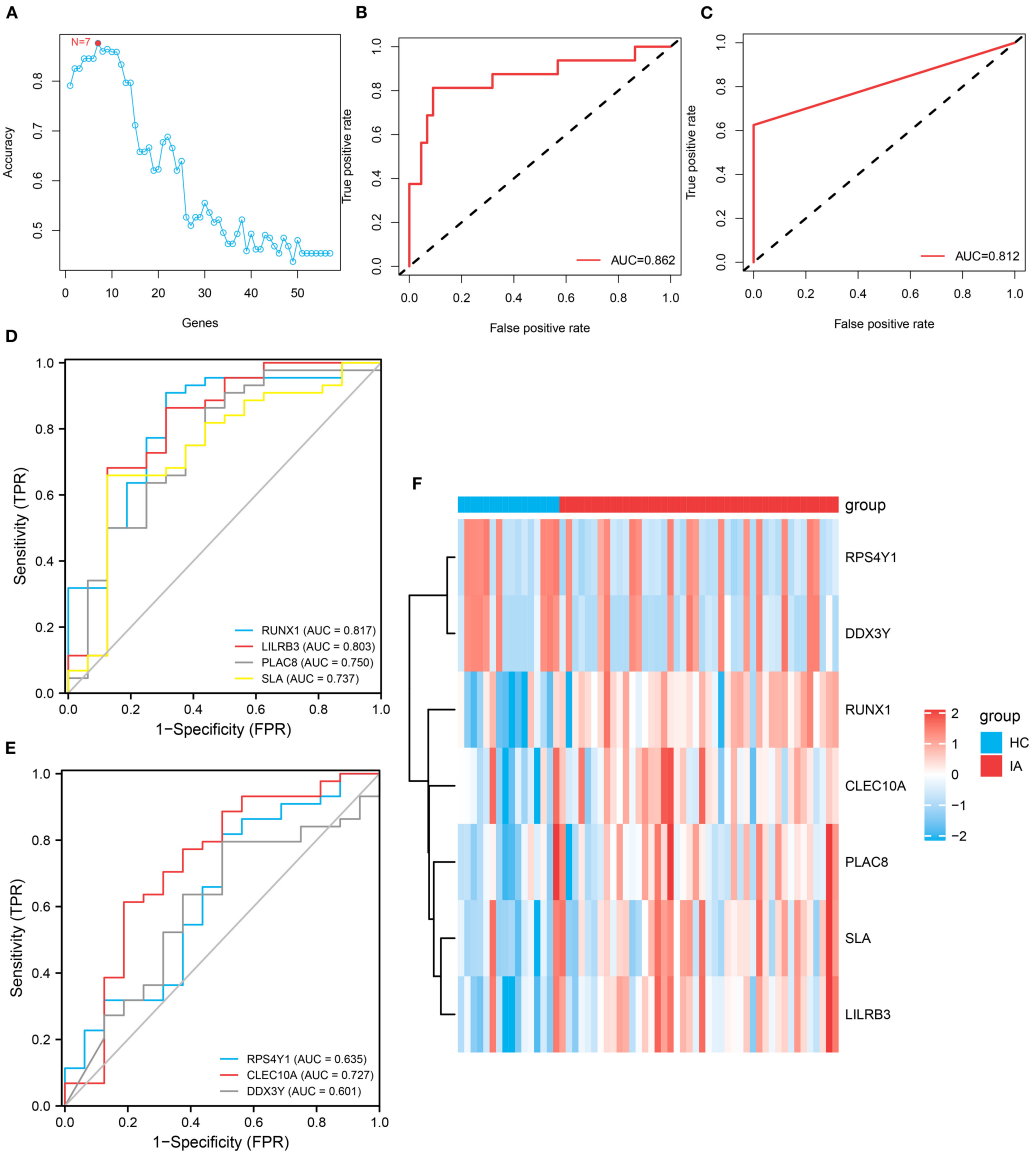
Construction and variable selection of the intracranial aneurysm prediction model using the Support Vector Machines–Recursive Feature Elimination (SVM–RFE) algorithm. **(A)** Broken line graph of the number of genes identified using the SVM–RFE model and the accuracy of the model. Hence, the highest accuracy can be achieved using the SVM model constructed from seven genes. **(B)** Receiver operating characteristic (ROC) curve based on the SVM model constructed from these seven genes, with an area under the curve (AUC) value of 0.862. **(C)** Validation of this seven-gene SVM model by ROC analysis with an external cohort. **(D,E)** ROC curves showing the AUCs of *RUNX1, LILRB3, PLAC8, SLA, RPS4Y1, CLEC10A*, and *DDX3Y*. **(F)** Heatmap depicts the expression distribution of these seven genes in the bulk RNA of IA.

In total, 57 genes were identified as shared DEGs in monocytes/macrophages from AAA and IA (Figure 4G). These DEGs were then further screened by applying SVM–RFE to the bulk RNA-seq dataset from IA. The variable screening results of the SVM–RFE algorithm showed that the IA prediction model constructed from seven gene features (*RPS4Y1, DDX3Y, RUNX1, CLEC10A, PLAC8, SLA*, and *LILRB3*) had the highest accuracy (Figure 5A), with an area under the curve (AUC) of 0.862 (Figure 5B). This seven-gene SVM model was validated by ROC analysis with the GSE13353 dataset as an external cohort (AUC: 0.812) (Figure 5C). Figures 5D,E depict the ROC curves for each of the seven genes (AUC: 0.601–0.817). The above studies suggest that these genes (*RPS4Y1, DDX3Y, RUNX1, CLEC10A, PLAC8, SLA*, and *LILRB3*) can predict the prevalence of IA. Figure 5F depicts the expression distribution of these seven genes in the bulk RNA of IA. By analyzing the heat map, *RUNX1, CLEC10A, PLAC8, SLA*, and *LILRB3* were found to be highly expressed in IA, while *RPS4Y1* and *DDX3Y* were lowly expressed in IA.

### *RUNX1* As a Hub Gene in IA Progression Determined via Network Analysis and Intersection Analysis

**Figure 6:**
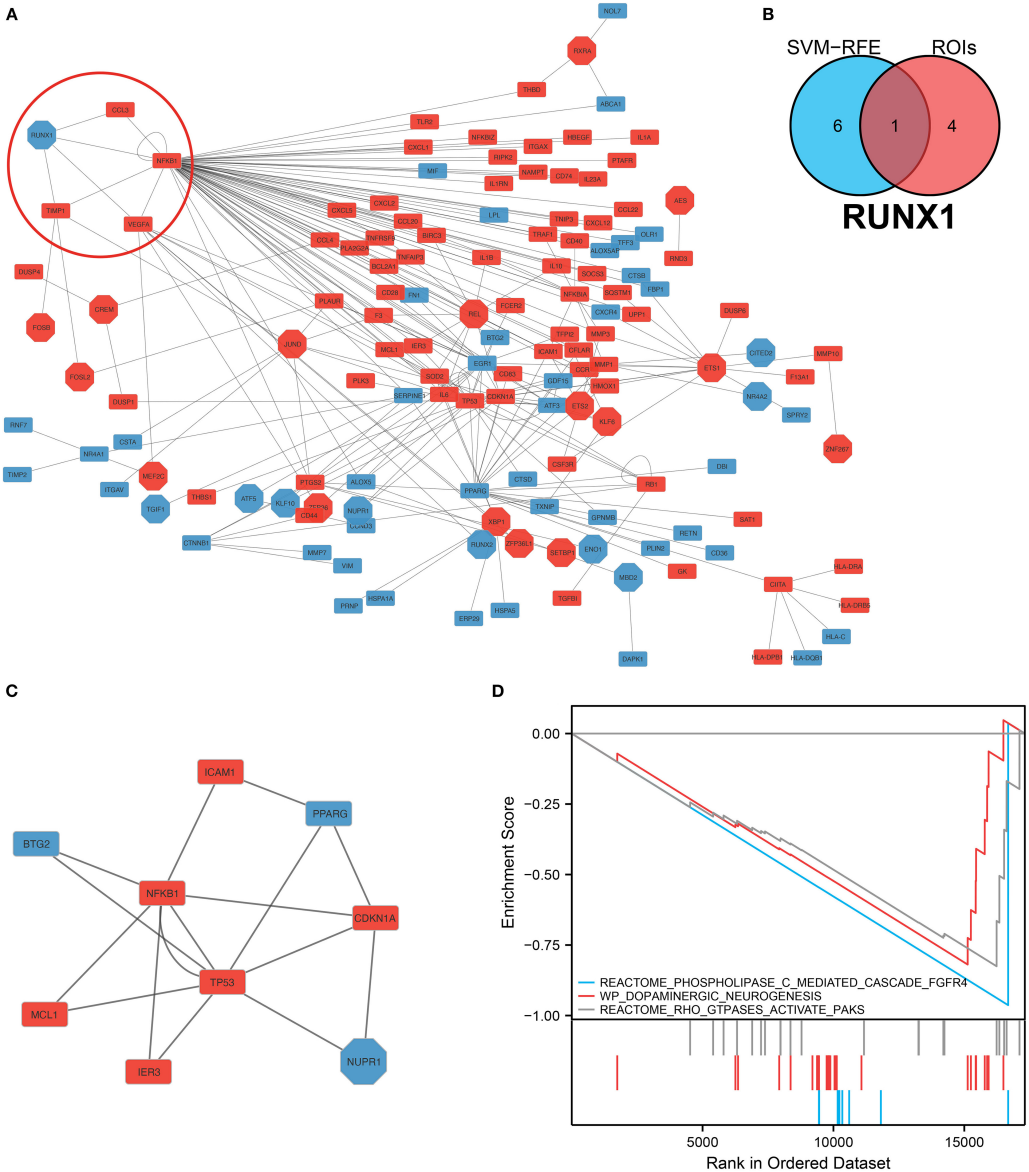
Construction of the protein–protein interaction network and hub gene screening. **(A)** TF-mRNA protein–protein interaction network in monocytes/macrophages. The red circles denote the *RUNX1* and target genes. **(B)** Intersection analysis of the seven genes screened using the SVM–RFE model, and these five regions of interest yielded the hub gene *RUNX1*. **(C)** Sub-protein–protein interaction network showing that *NFKB1*, which is regulated by *RUNX1*, is the hub gene in this PPI network. **(D)** Gene Set Enrichment Analysis of *RUNX1* at the single-cell level.

We analyzed and constructed the TF regulatory network in monocytes and macrophages (Figure 6A). In addition, the intersection analysis of the seven genes and five GOIs used to build the IA prediction model revealed that *RUNX1* was the hub gene (Figure 6B). In the PPI network, *RUNX1* was responsible for regulating four downstream molecules. According to the core sub-network of this PPI network, *NFKB1*, regulated by *RUNX1*, is the hub gene of the entire network (Figure 6C). Therefore, *RUNX1* was identified as a hub gene in the IA process by network analysis and crossover analysis.

### Correlation Between *RUNX1* and IA Progression

**Figure 7:**
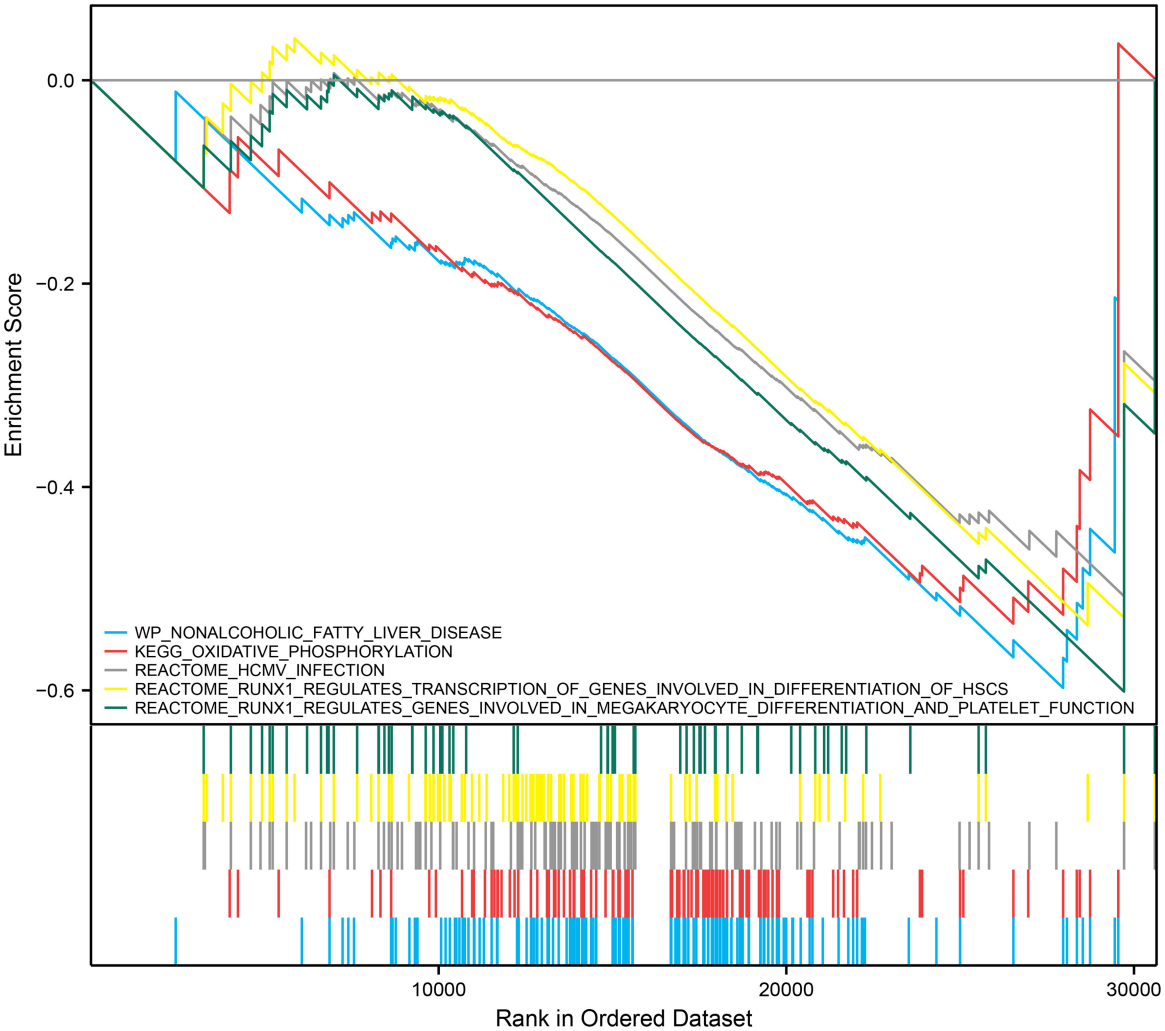
*RUNX1* GSEA at the bulk RNA level.

We performed GSEA analysis of monocytes/macrophages in the single cell dataset. GSEA showed phospholipase C-mediated cascade FGFR4, dopaminergic neurogenesis, and RHO GTPases activate PAKs were downregulated in monocytes/macrophages upregulated with *RUNX1* (Figure 6D). Based on GSE122897, GSEA was also further performed to explore the functional pathways enriched for *RUNX1* at the individual level (Figure 7). Moreover, it revealed that in samples with upregulated *RUNX1* expression, nonalcoholic fatty liver disease, oxidative phosphorylation, *RUNX1* regulates the transcription of genes involved in the differentiation of HSCs and those involved in megakaryocyte differentiation, and platelet function pathway *RUNX1* regulates genes involved in megakaryocyte differentiation and platelet function. Therefore, *RUNX1* plays a complex role in IA progression. Further, it might be involved in IA progression via the regulation of HSC differentiation and platelet production and might play a potential regulatory role in oxidative phosphorylation.

## Discussion

From cellular data, it is evident that *RUNX1* contributes to the development of IA via its role in regulating *NFKB1* in monocytes/macrophages within the hypertensive microenvironment. GSEA revealed that *RUNX1* was associated with IA progression by regulating hematopoietic stem cell differentiation and platelet production.

In this study, scRNA-seq data were used to examine the transcriptional patterns of IA at the single-cell level. The risk mRNAs of IA were screened, and predictive models were established using the combined bulk RNA-seq data. Further, the IA-related TF-gene regulatory network was constructed. *RUNX1* was identified as the hub gene, and it regulated four genes. And *NFKB1* was found to be regulated by *RUNX1*. This finding was consistent with that of previous studies showing that NF-κB was involved in IA progression (44, 45). And we first identified the mechanism at the single-cell level.

*RUNX1* was significantly expressed in the IA group (P < 0.001). GSEA revealed that phospholipase C-mediated cascade FGFR4, dopaminergic neurogenesis, and RHO GTPases activate PAKs were downregulated in monocytes/macrophages cells where *RUNX1* was upregulated. Thus, *RUNX1* plays a complex role in IA progression, possibly via the regulation of hematopoietic stem cell differentiation and platelet production. *RUNX1* might be a hub gene between hypertension, IA, and DEGs in monocytes. Previous studies have found that *RUNX1* is involved in hypertension progression to some extent. Further, it is an important hematopoietic transcription factor associated with thrombocytopenia and impaired platelet activation response and is correlated with vascular disease progression (46). *RUNX1* is involved in endothelial cells and hematopoietic processes, thereby affecting endothelial function and inflammatory changes, and the abnormal expression of *RUNX1* can induce hypertension (47). An experimental study has shown that the inhibition of *RUNX1* expression decreases pulmonary hypertension progression in mice (47). In addition, it reduces vascular remodeling and macrophage recruitment, which play essential roles in hypertension progression (48, 49). Hypertension is a significant risk factor for IA progression.

The role of *RUNX1* identified in related studies may be relevant to IA. *RUNX1* is involved in regulating endothelial cell function and is a key transcriptional regulator of the conversion of endothelial to hematopoietic cells (47, 50). An early feature of IA is endothelial cell dysfunction and degeneration (51). Lilly et al. found that SOX7 interacted with *RUNX1* at the protein level and inhibited the transcriptional activity of *RUNX1*, thereby regulating the conversion of endothelial to hematopoietic cells and maintaining arterial endothelium stability (52). *RUNX1* can directly or indirectly regulate signal transduction pathways such as the TGF-β signaling pathway and the Wnt signaling pathway (53). TGF-β is associated with cerebral edema after subarachnoid hemorrhage and can be used as a prognostic indicator (54). Therefore, *RUNX1* in the TGF-β signaling pathway may be correlated with the progression and prognosis of IA. Based on the functions played by *RUNX1* in the progression of hypertension and IA, we hypothesized that *RUNX1* promotes hypertension progression combined with IA.

A population-wide genomic study showed that *NFKB1* is a susceptibility gene for primary hypertension (54). *NFKB1* is involved in hypertension progression by affecting vascular endothelial function via the regulation of downstream NOS3 gene expression (55). It also promotes oxidative stress injury in gestational hypertensive mice, which contributes to hypertension pathogenesis (56–58). In addition, NF-κB is involved in the IA progression via several pathways. The activation of NF-κB in signaling pathways upregulates MCP-1 expression and participates in the apoptotic process in vascular smooth muscle cells. This phenomenon further reduces the elasticity of the cerebral vascular wall, making it less able to adapt to altered hemodynamics, promotes IA progression, and increases the risk of rupture (59–61). NF-κB elevates the transcription of various pro-inflammatory genes such as *COX-2, CCL-2, MMP*, and *iNOS*, and the inflammatory response is involved in the progression and rupture of IA (62, 63). Moreover, macrophage infiltration and NF-κB activation are reduced if macrophages are specifically absent or in the mutants of the NF-κB inhibitory protein. To the best of our knowledge, this study first identified the role of *RUNX1* in monocytes/macrophages at the single-cell level in IA progression among patients with hypertension via the regulation of *NFKB1*.

The purpose of this study was to investigate the potential mechanisms of IA induction by monocytes and macrophages in the hypertensive microenvironment at the single-cell level using bioinformatics. *RUNX1* and its regulated *NFKB1* were identified as hub genes. *RUNX1* may play a role in the progression of IA by regulating abnormalities in hematopoietic stem cell differentiation and platelet production. Identification of these two genes is an important step in understanding IA mechanisms in patients with hypertension. Moreover, it can facilitate further research about potential targets for preventing and reducing the risk of IA in patients with hypertension. There was no single-cell dataset available for IA assessment; therefore, the single-cell dataset (GSE166676) for AAA assessment was used instead. The dataset should be expanded in the future. Finally, neither cellular nor animal tests were conducted to investigate the signaling pathways of the identified hub genes. Further work is required to corroborate these findings.

## Conclusion

*RUNX1* in monocytes/macrophages is associated with the development of IA via the expression of *NFKB1* among patients with hypertension. This potential role can lay the foundation for the further identification of molecular mechanisms underlying IA progression in patients with hypertension and can provide data about treatment targets in IA among patients with hypertension.

## Data Availability Statement

Publicly available datasets were analyzed in this study. This data can be found here: National Center for Biotechnology Information (NCBI) Gene Expression Omnibus (GEO), https://www.ncbi.nlm.nih.gov/geo/, GSE75360 data set and GSE122897.

## Ethics Statement

Ethical review and approval was not required for the study on human participants in accordance with the local legislation and institutional requirements. Written informed consent from the patients/participants or patients/participants' legal guardian/next of kin was not required to participate in this study in accordance with the national legislation and the institutional requirements.

## Author Contributions

YL: software, validation, formal analysis, data curation, and writing—original draft. ZZ: software, validation, formal analysis, supervision, data curation, and writing—original draft. DL: conceptualization, methodology, supervision, writing—review and editing, and project administration. All authors contributed to the article and approved the submitted version.

## Funding

This work was funded by Project of Ningxia Medical University (XZ2021025 to YL). This work was also supported by the Natural Science Foundation of Shandong Province (A study on the mechanism of temporal lobe epilepsy comorbid anxiety based on negative emotional information processing in amygdala activation and changes in the effective link of brain function. No. ZR2019BH043).

## Conflict of Interest

The authors declare that the research was conducted in the absence of any commercial or financial relationships that could be construed as a potential conflict of interest.

## Publisher's Note

All claims expressed in this article are solely those of the authors and do not necessarily represent those of their affiliated organizations, or those of the publisher, the editors and the reviewers. Any product that may be evaluated in this article, or claim that may be made by its manufacturer, is not guaranteed or endorsed by the publisher.

## Abbreviations

AAA: Abdominal aortic aneurysm
IA: Intracranial aneurysm
*RUNX1*: Runt-related transcription factor 1
SAH: Subarachnoid hemorrhage
SVM-RFE: Support Vector Machines–Recursive Feature Elimination algorithm.
