# Supplementary material for: Intracranial Aneurysms Induced by RUNX1 Through Regulation of NFKB1 in Patients With Hypertension-An Integrated Analysis Based on Multiple Datasets and Algorithms
Source: Front Neurol. 2022 May 17;13:877801. doi: 10.3389/fneur.2022.877801 (PMC9152011; doi:10.3389/fneur.2022.877801)
Supplement: Supplementary file 1 [file Data_Sheet_1.ZIP › 4.1_go.kegg/GO_barplot.down.pdf]

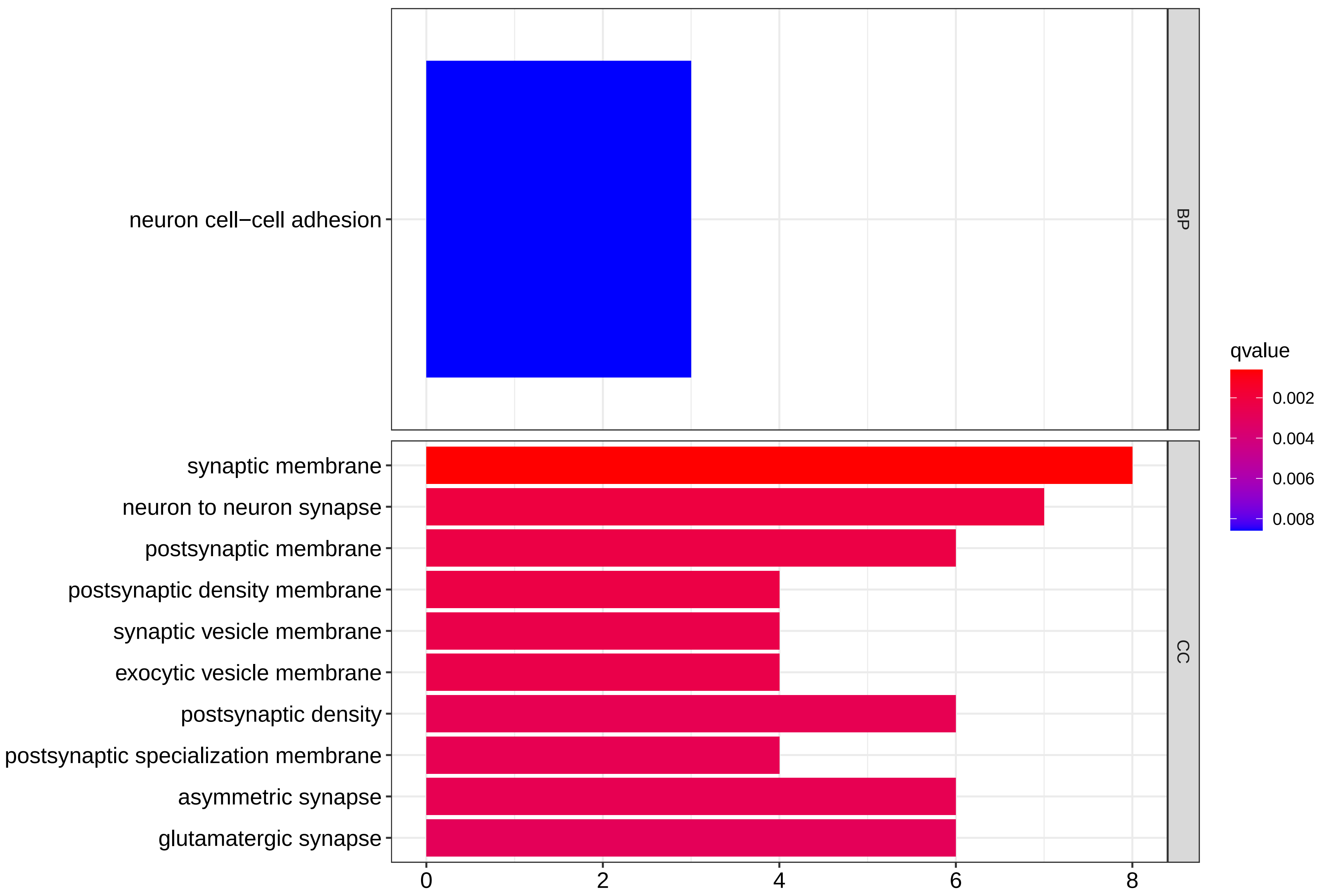
neuron cell−cell adhesion
BP
synaptic membrane
neuron to neuron synapse
postsynaptic membrane
postsynaptic density membrane
synaptic vesicle membrane
exocytic vesicle membrane
postsynaptic density
postsynaptic specialization membrane
asymmetric synapse
glutamatergic synapse
CC
0
2
4
6
8
qvalue
0.002
0.004
0.006
0.008
