# Supplementary material for: Intracranial Aneurysms Induced by RUNX1 Through Regulation of NFKB1 in Patients With Hypertension-An Integrated Analysis Based on Multiple Datasets and Algorithms
Source: Front Neurol. 2022 May 17;13:877801. doi: 10.3389/fneur.2022.877801 (PMC9152011; doi:10.3389/fneur.2022.877801)
Supplement: Supplementary file 1 [file Data_Sheet_1.ZIP › 4.1_go.kegg/KEGG_bubble.down.pdf]

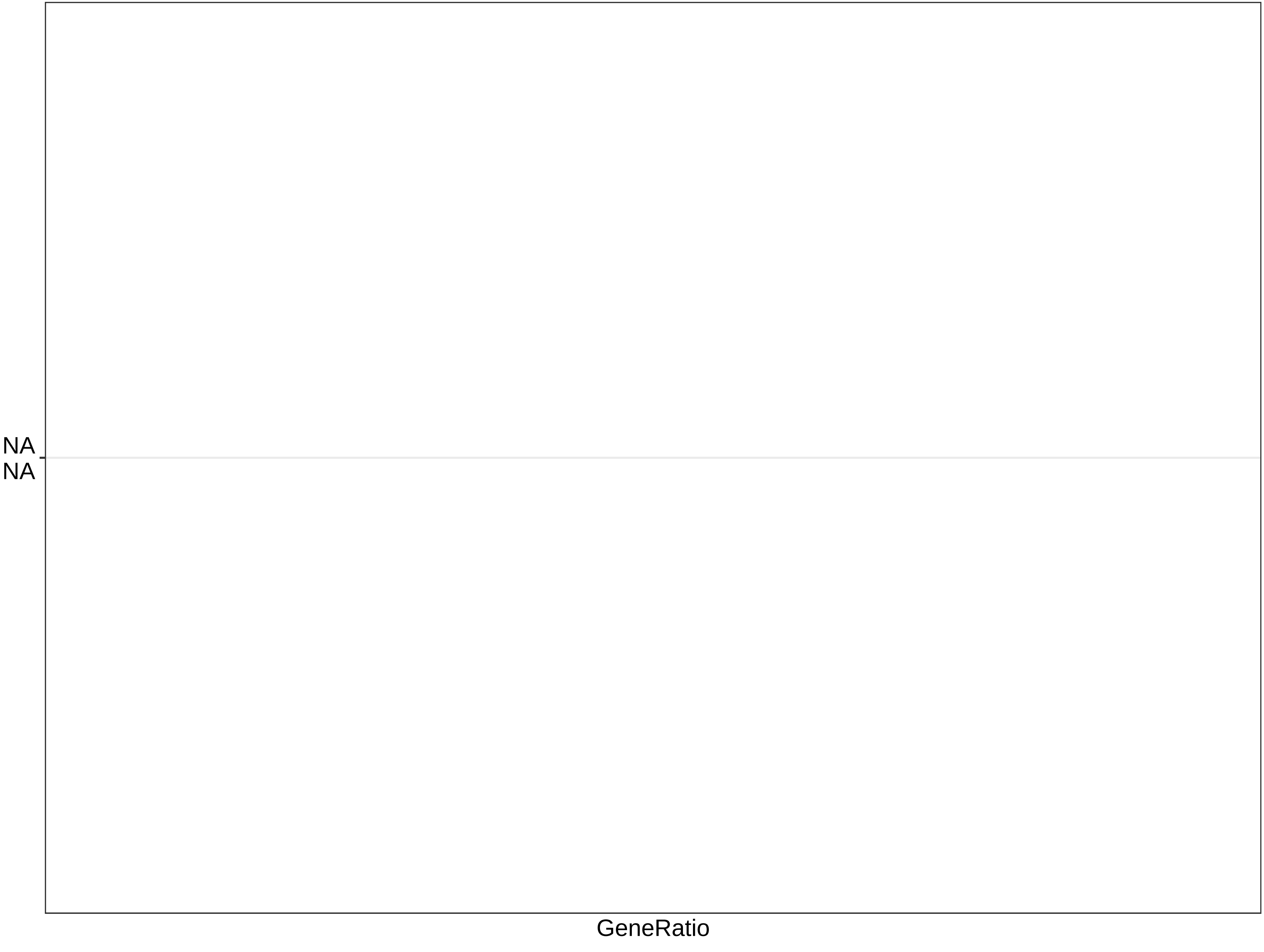
NA
NA
GeneRatio
