# Supplementary figures and images for: Intracranial Aneurysms Induced by RUNX1 Through Regulation of NFKB1 in Patients With Hypertension-An Integrated Analysis Based on Multiple Datasets and Algorithms
Source: Front Neurol. 2022 May 17;13:877801. doi: 10.3389/fneur.2022.877801 (PMC9152011; doi:10.3389/fneur.2022.877801)

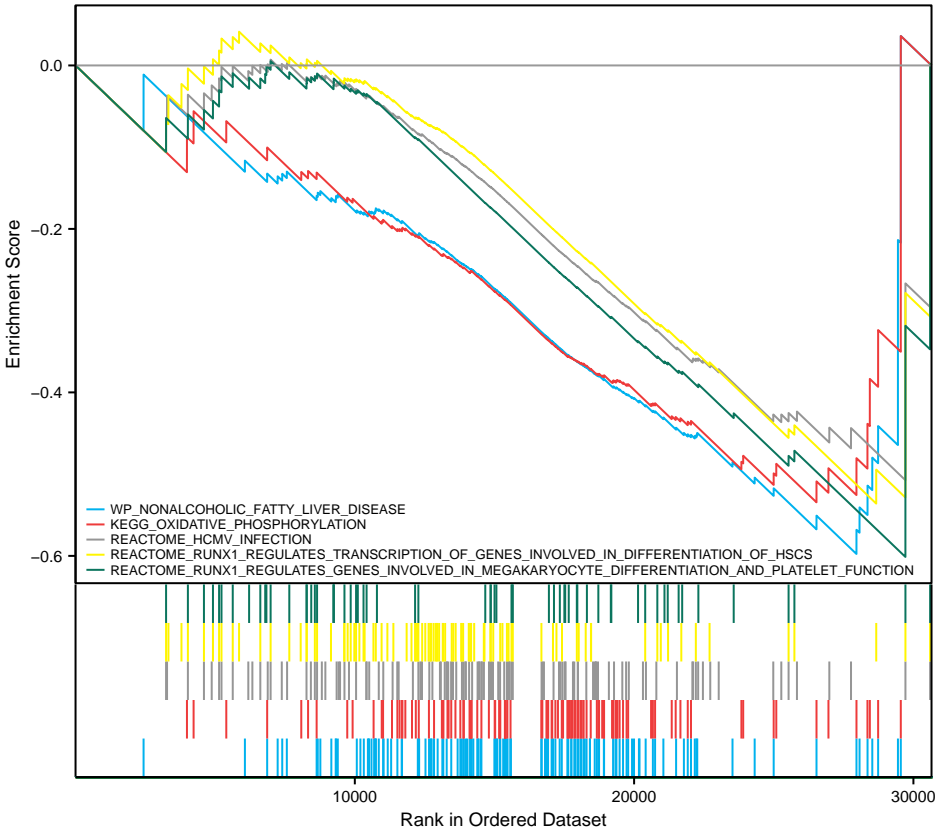

Supplement: Supplementary file 1 [file Data_Sheet_1.ZIP › 10_ia_gsea/RUNX1/plot/GSEA可视化_2022-01-10_00_59_24.pdf]

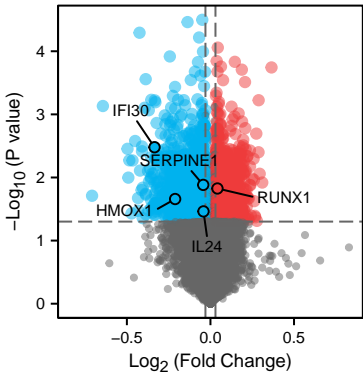

Supplement: Supplementary file 1 [file Data_Sheet_1.ZIP › 1_HTdiff/plot/火山图_2022-01-05_20_31_36.pdf]

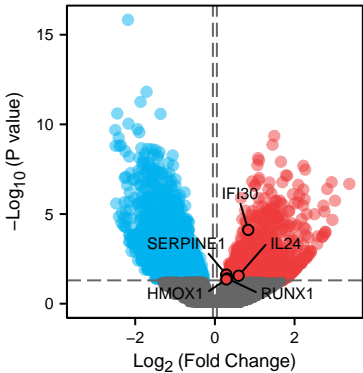

Supplement: Supplementary file 1 [file Data_Sheet_1.ZIP › 2_IAdiff/plot/火山图_2022-01-05_20_36_44.pdf]

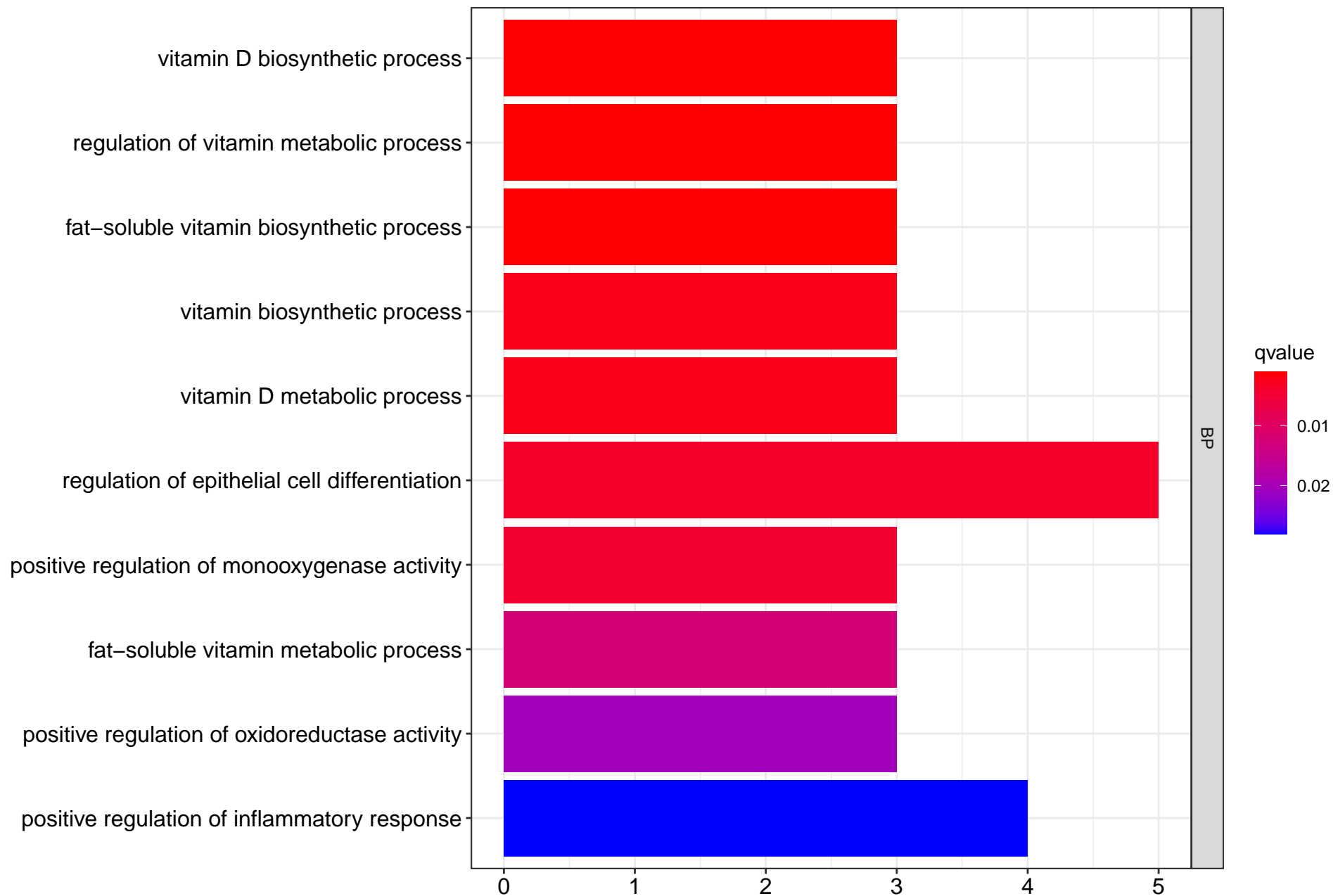

Supplement: Supplementary file 1 [file Data_Sheet_1.ZIP › 4.1_go.kegg/GO_barplot.up.pdf]

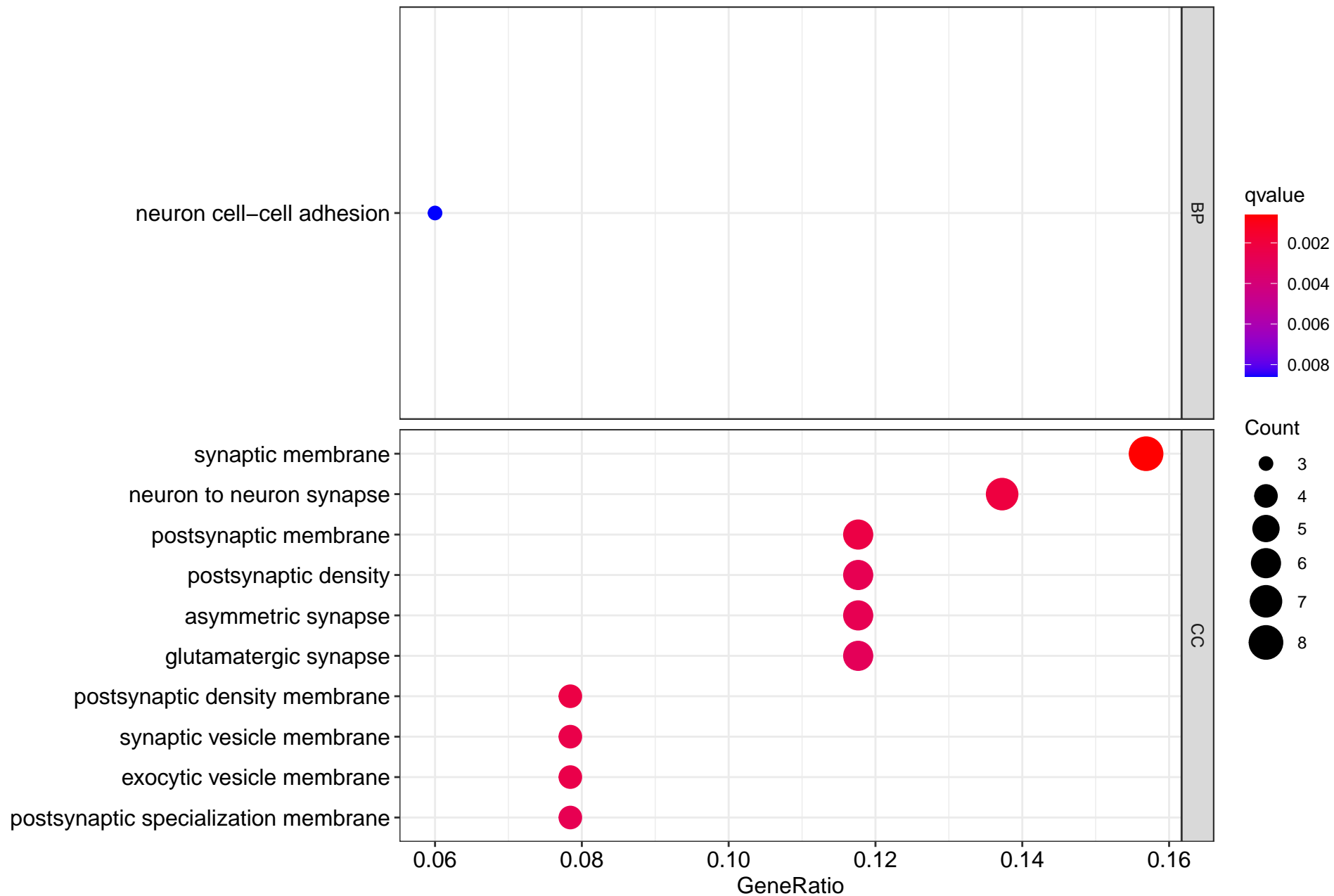

Supplement: Supplementary file 1 [file Data_Sheet_1.ZIP › 4.1_go.kegg/GO_bubble.down.pdf]

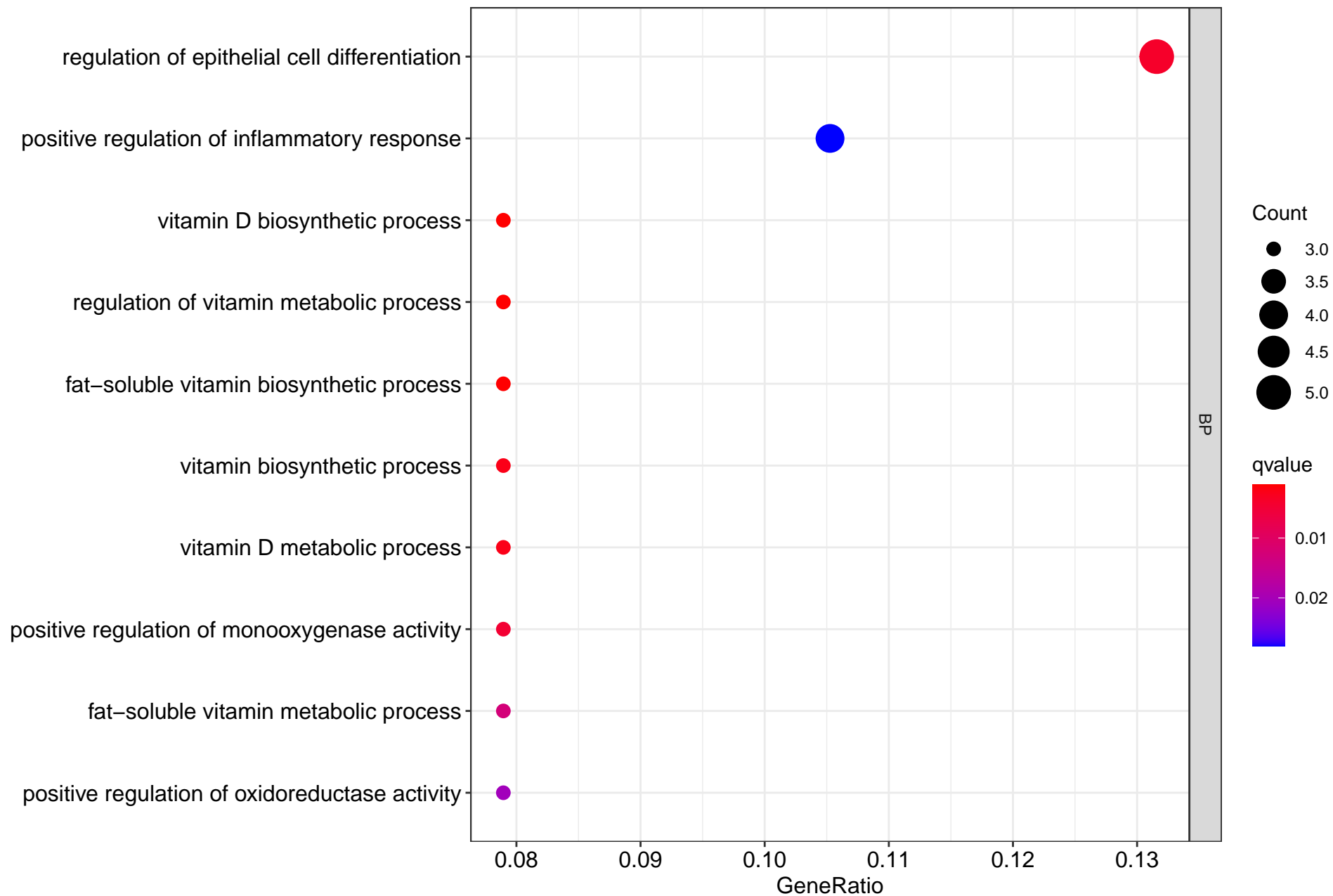

Supplement: Supplementary file 1 [file Data_Sheet_1.ZIP › 4.1_go.kegg/GO_bubble.up.pdf]

Cytokine–cytokine receptor interaction

HIF–1 signaling pathway

qvalue

0.006306899

0

2

4

6

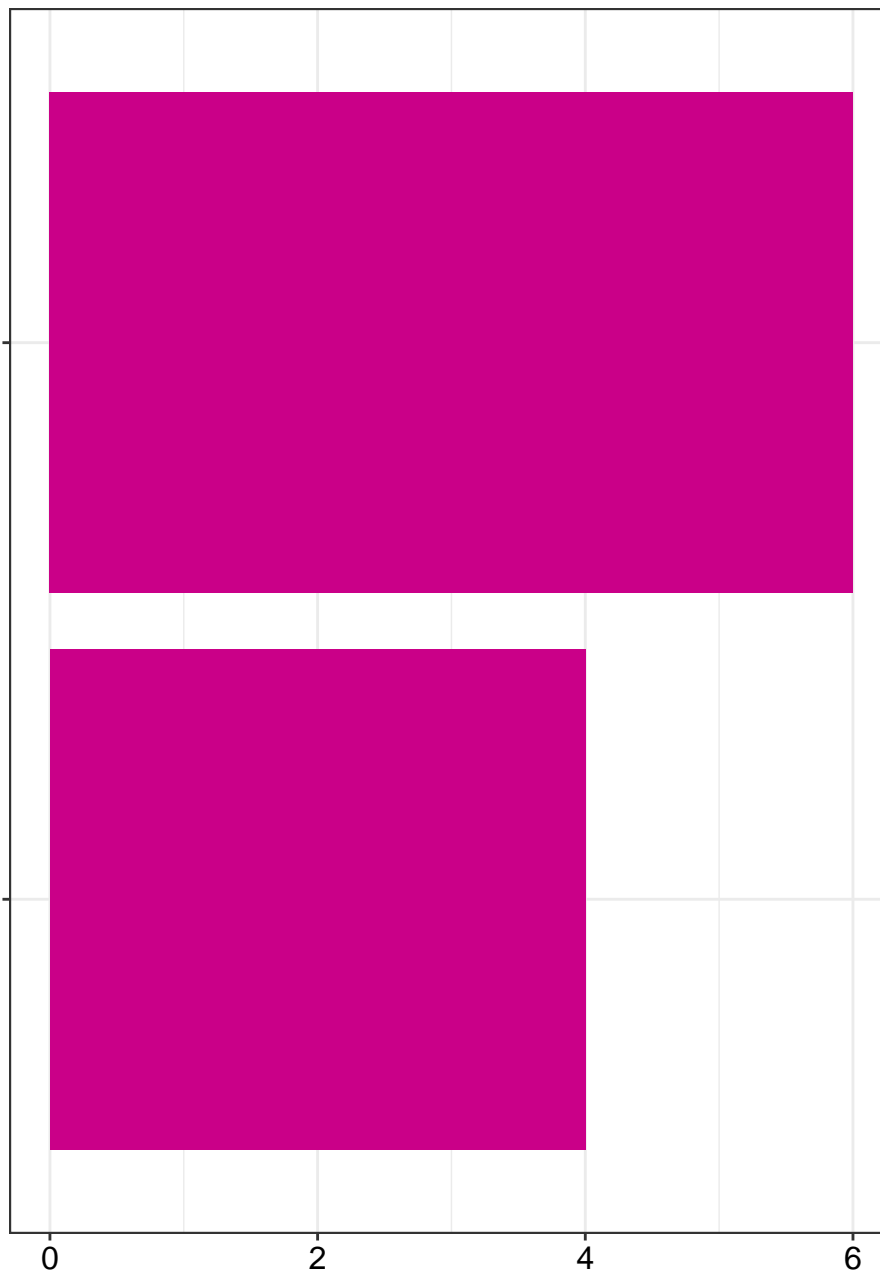

Supplement: Supplementary file 1 [file Data_Sheet_1.ZIP › 4.1_go.kegg/KEGG_barplot.up.pdf]

Cytokine–cytokine receptor interaction

HIF–1 signaling pathway

Count

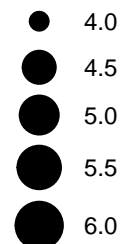

qvalue

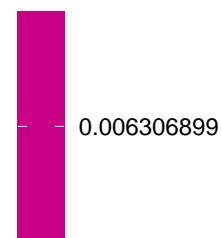

0.200

0.225  
GeneRatio

0.250

0.275

Supplement: Supplementary file 1 [file Data_Sheet_1.ZIP › 4.1_go.kegg/KEGG_bubble.up.pdf]

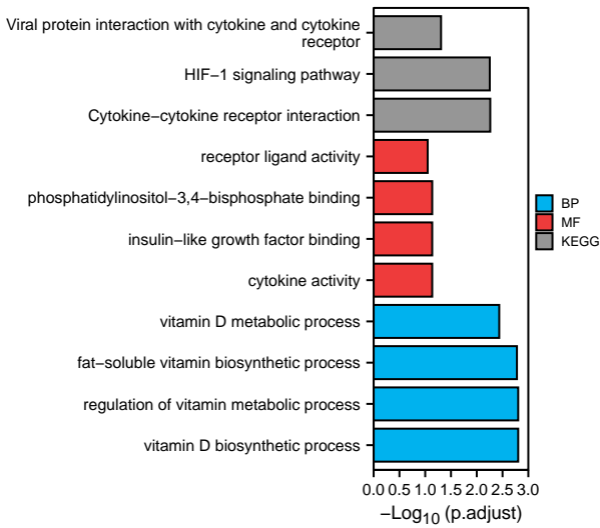

Supplement: Supplementary file 1 [file Data_Sheet_1.ZIP › 4.1_go.kegg/plot/GO_KEGG可视化_2022-01-05_21_29_58.pdf]

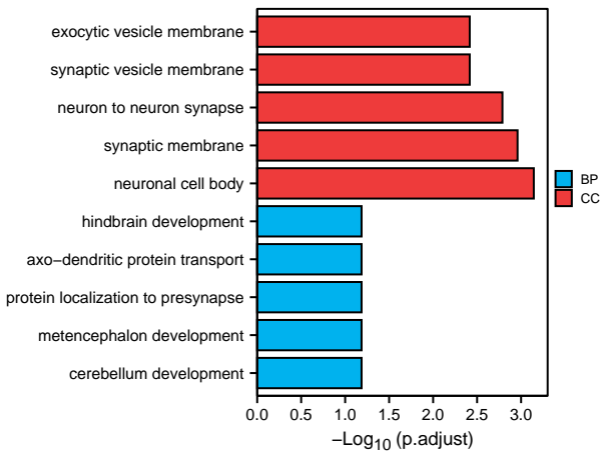

Supplement: Supplementary file 1 [file Data_Sheet_1.ZIP › 4.1_go.kegg/plot/GO_KEGG可视化_2022-01-05_21_30_22.pdf]

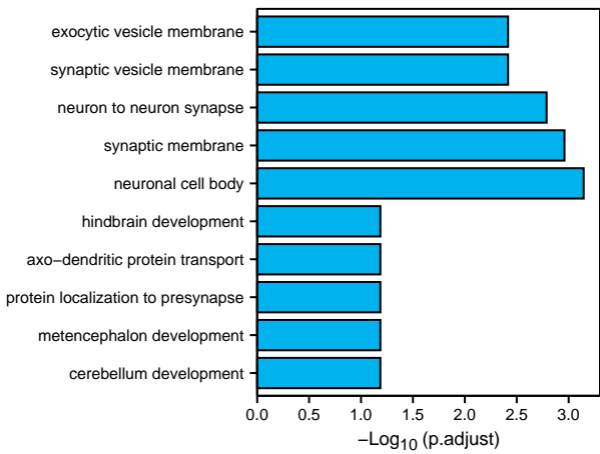

Supplement: Supplementary file 1 [file Data_Sheet_1.ZIP › 4.1_go.kegg/plot/GO_KEGG可视化_2022-01-05_21_32_11.pdf]

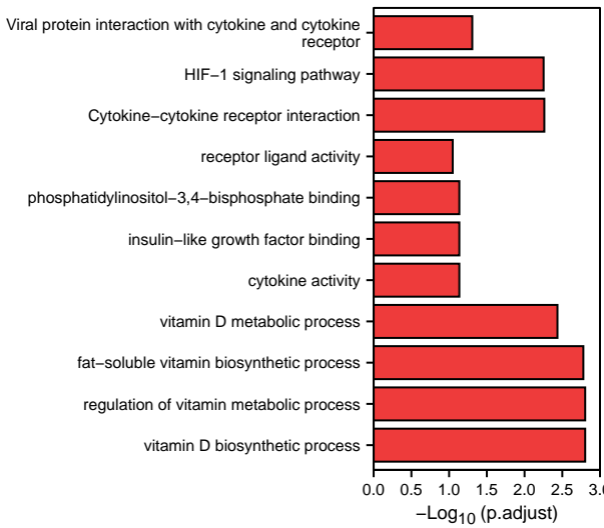

Supplement: Supplementary file 1 [file Data_Sheet_1.ZIP › 4.1_go.kegg/plot/GO_KEGG可视化_2022-01-10_19_36_11.pdf]

HT

2373

90

82

5

2932

52

730

IA

SingleCell

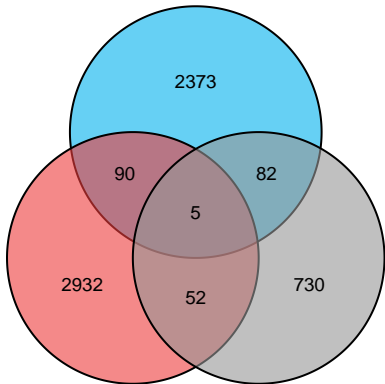

Supplement: Supplementary file 1 [file Data_Sheet_1.ZIP › 4_intersect/plot/维恩图_2022-01-05_20_18_16.pdf]

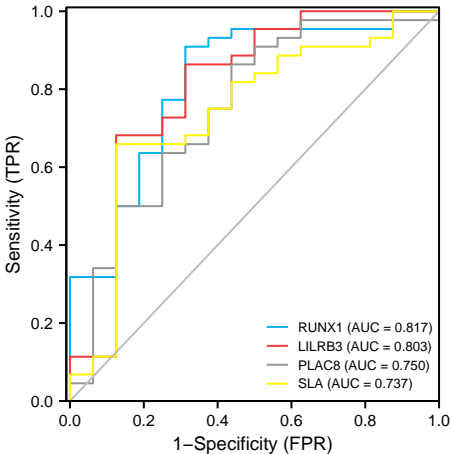

Supplement: Supplementary file 1 [file Data_Sheet_1.ZIP › 6_svm/plot2/诊断性ROC_2022-01-10_01_35_18.pdf]

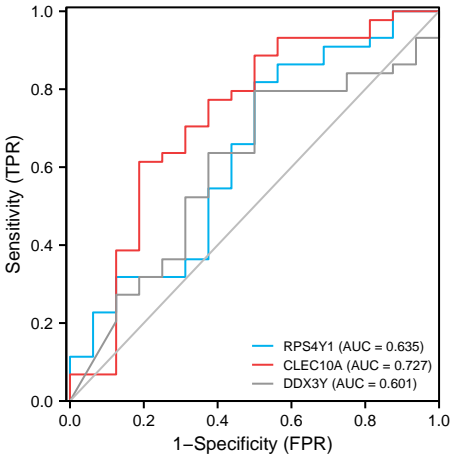

Supplement: Supplementary file 1 [file Data_Sheet_1.ZIP › 6_svm/plot2/诊断性ROC_2022-01-10_01_35_47.pdf]

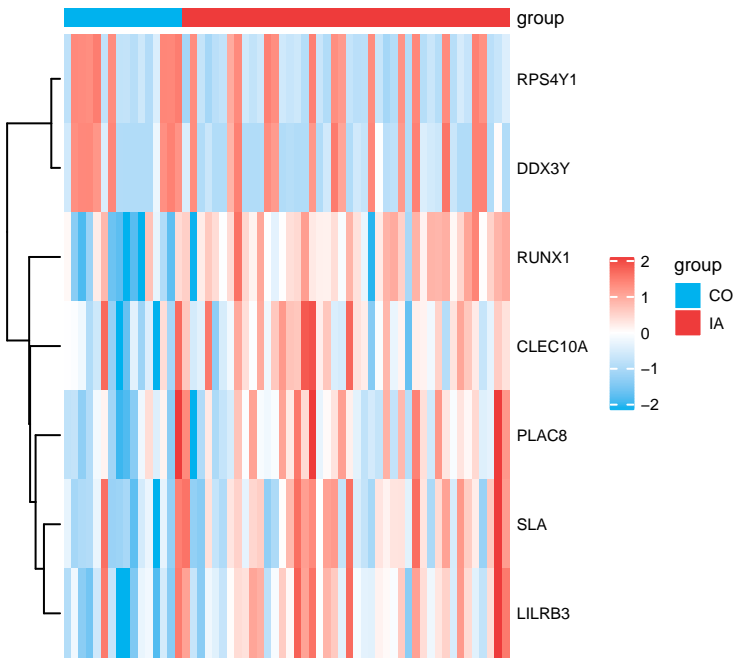

Supplement: Supplementary file 1 [file Data_Sheet_1.ZIP › 6_svm/plot/复杂热图_2022-01-10_01_27_00.pdf]

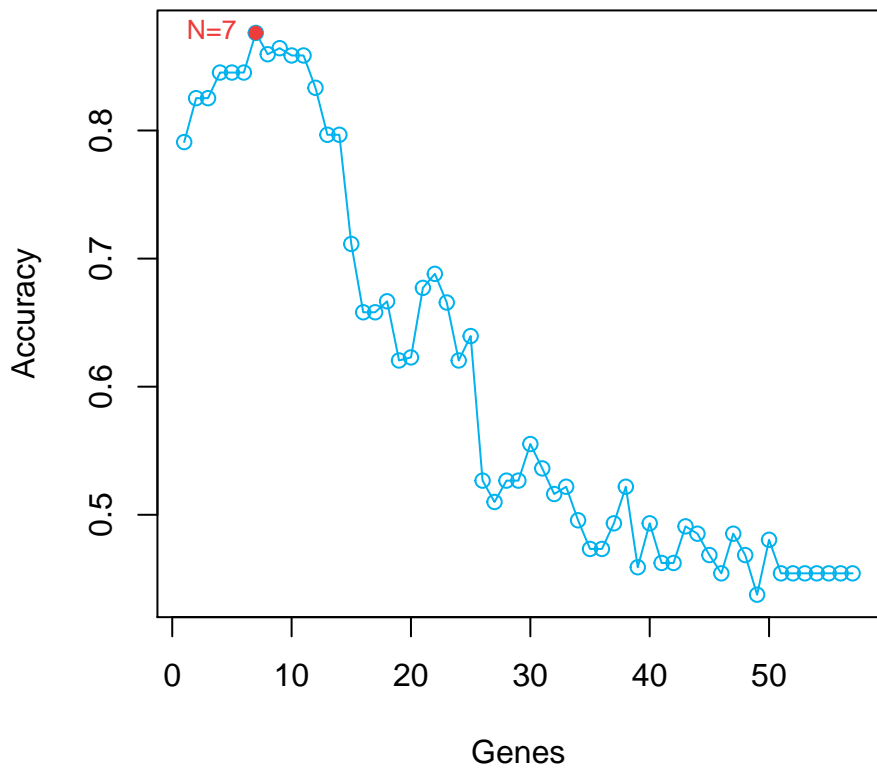

Supplement: Supplementary file 1 [file Data_Sheet_1.ZIP › 6_svm/svm-rfe.pdf]

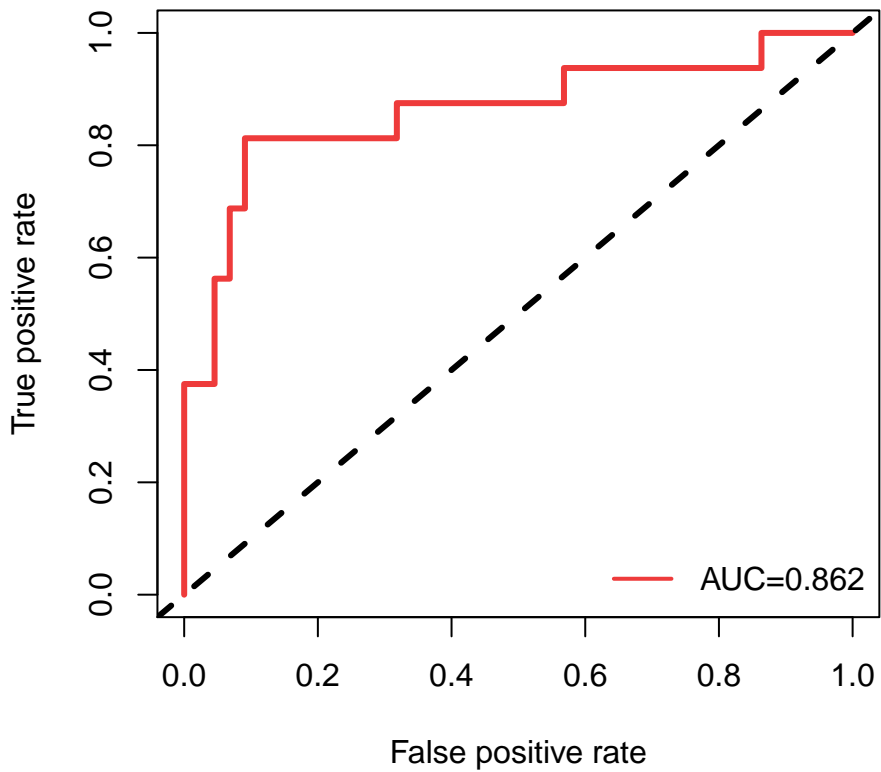

Supplement: Supplementary file 1 [file Data_Sheet_1.ZIP › 6_svm/svm.ROC.pdf]

SVM-RFE

ROIs

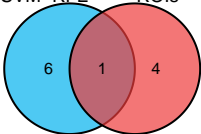

Supplement: Supplementary file 1 [file Data_Sheet_1.ZIP › 7_hub_gene/plot/维恩图_2022-01-10_14_06_20.pdf]

**AAA**

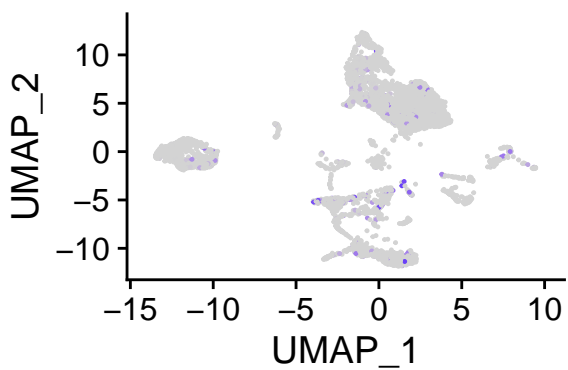

**NAC**

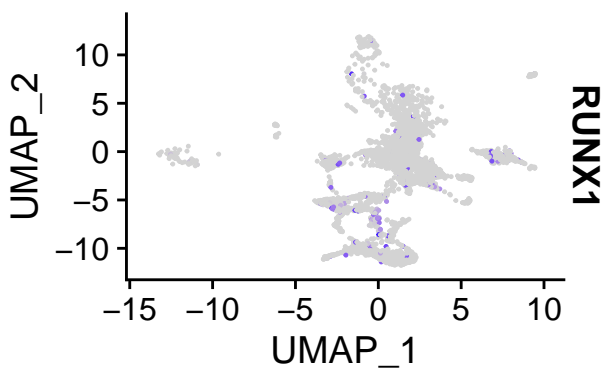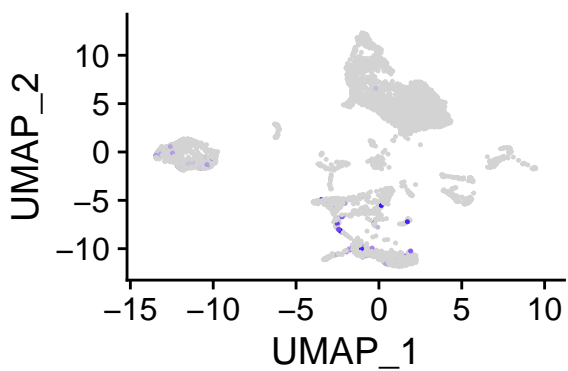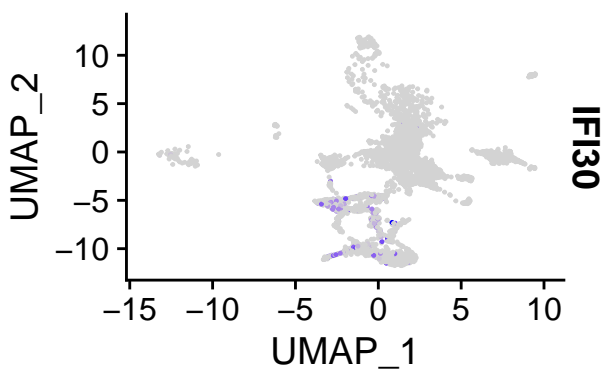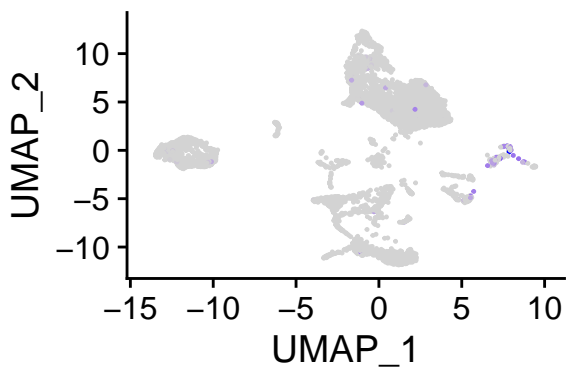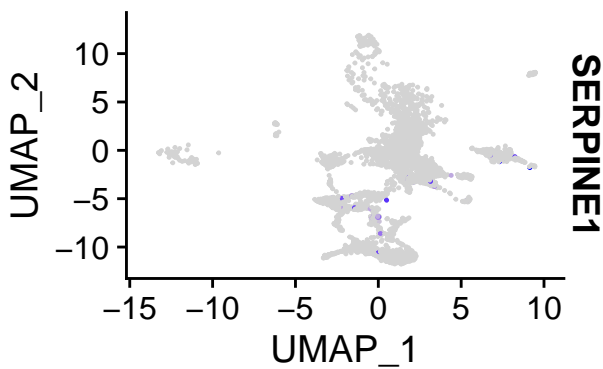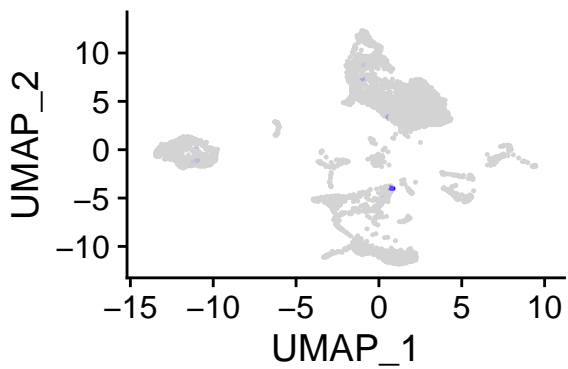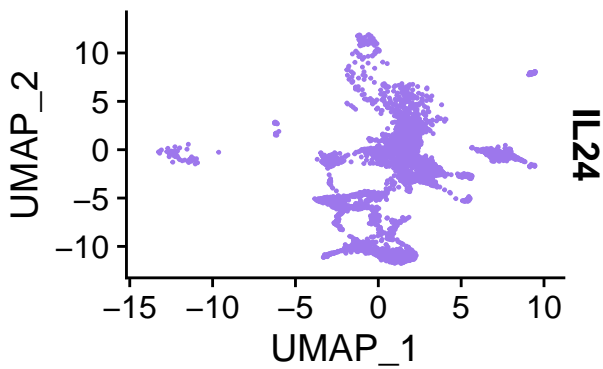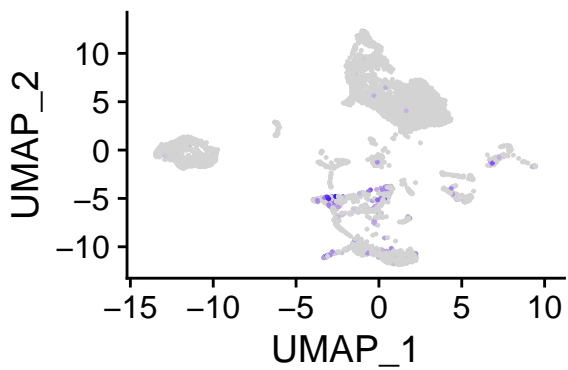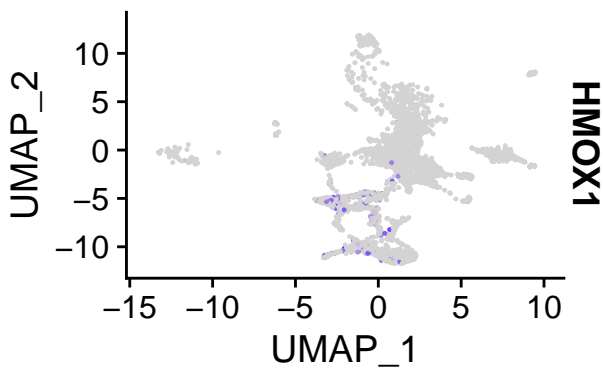

Supplement: Supplementary file 1 [file Data_Sheet_1.ZIP › 8_scRNA_mono/all_gene_FeaturePlot.pdf]

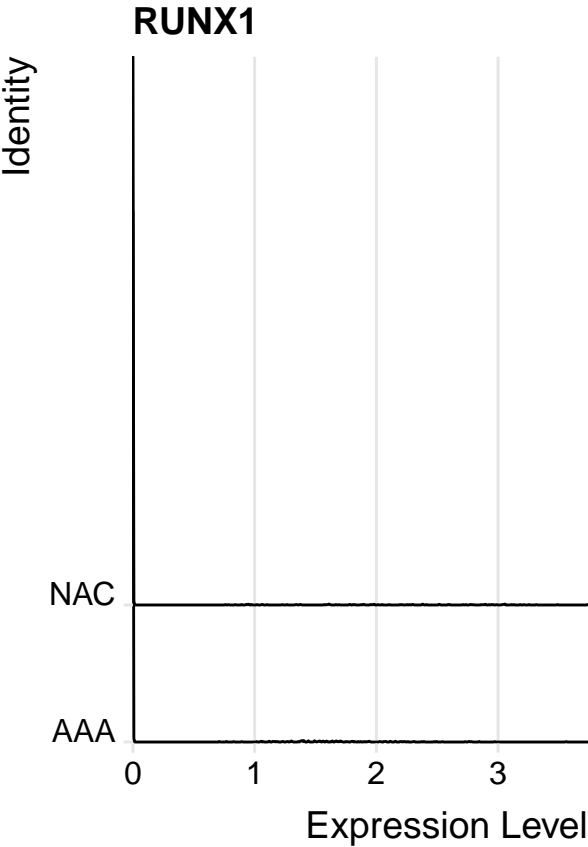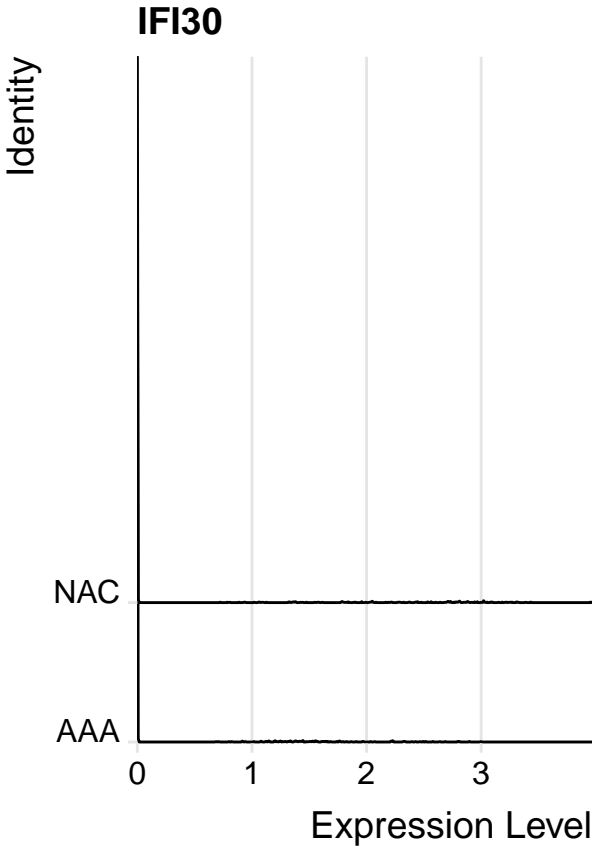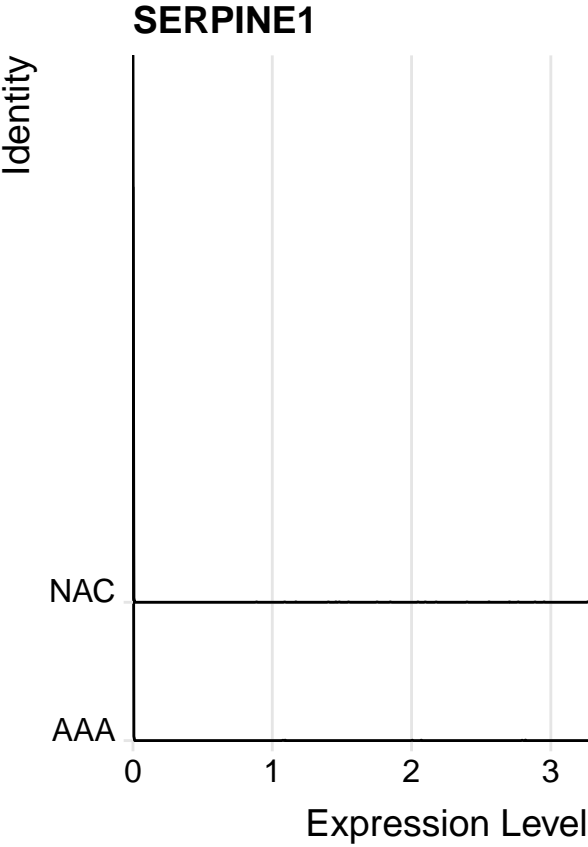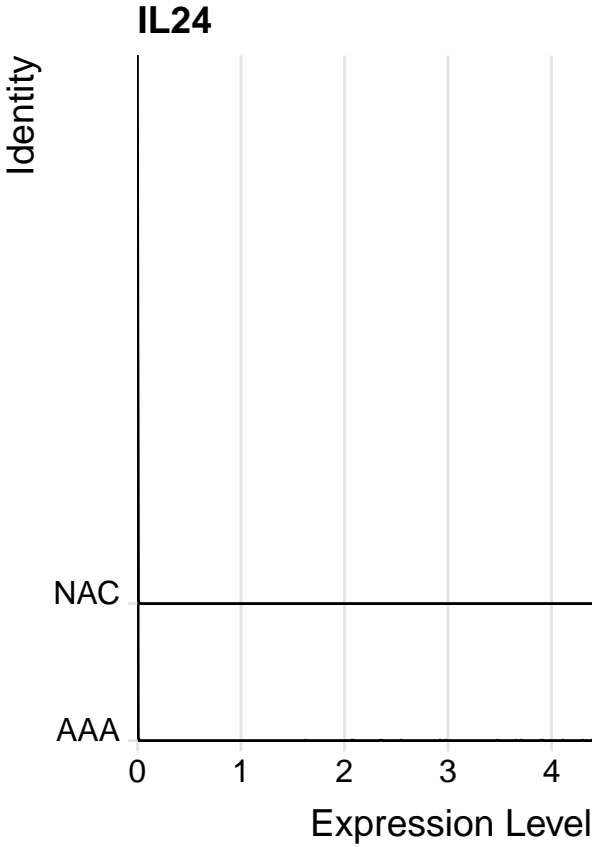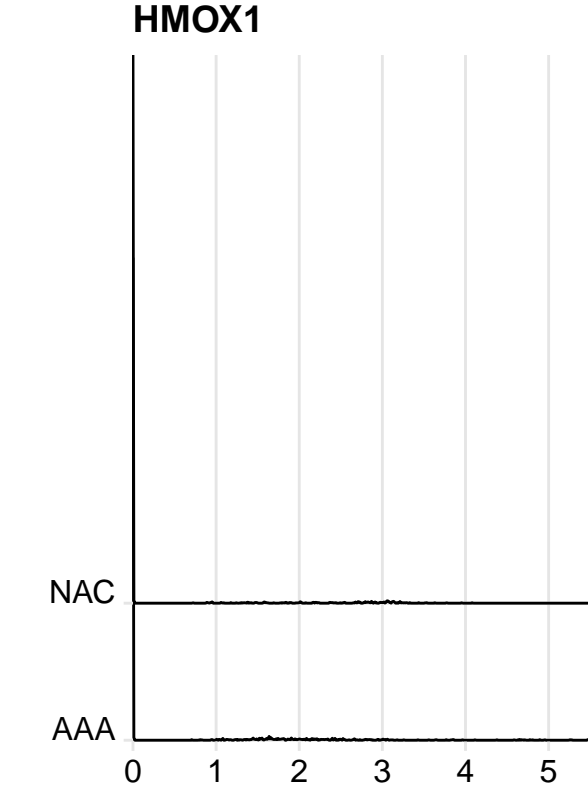

Supplement: Supplementary file 1 [file Data_Sheet_1.ZIP › 8_scRNA_mono/ann_marker_RidgePlot.pdf]

**RUNX1**

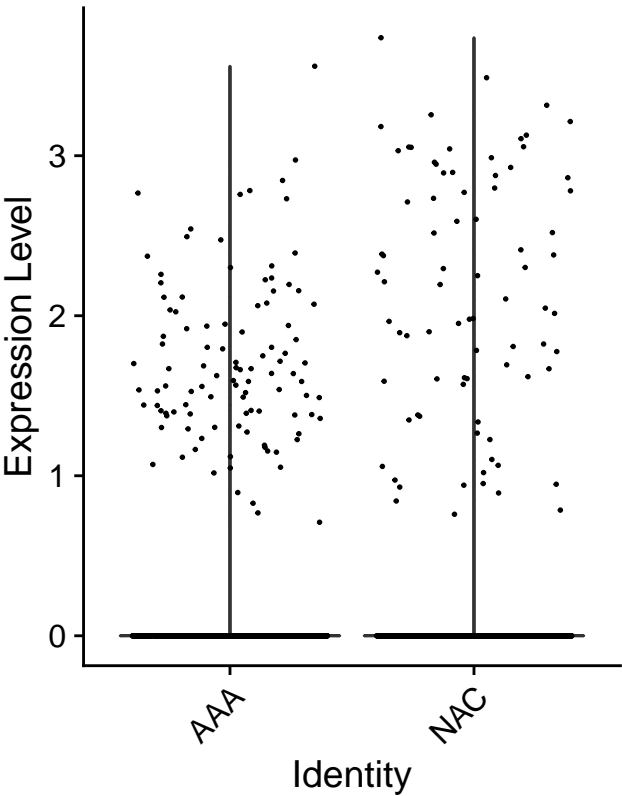

**IFI30**

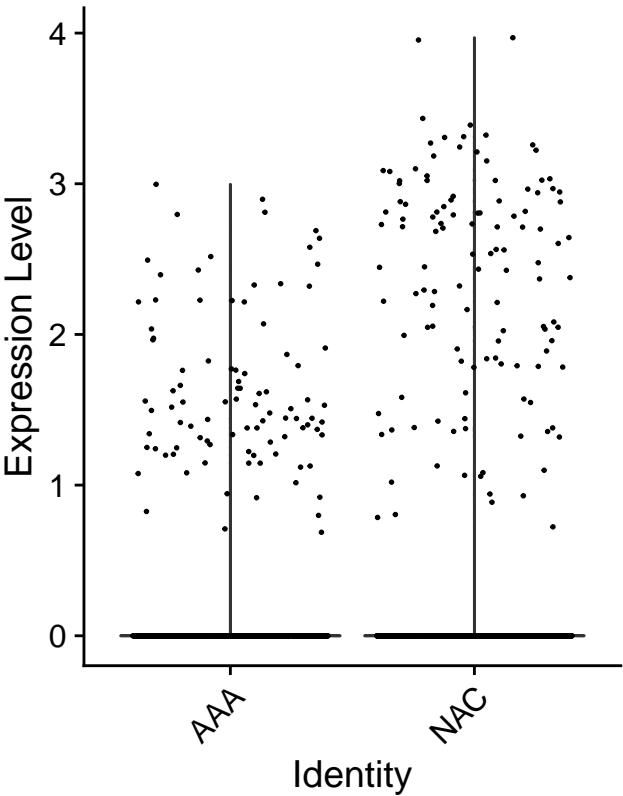

**SERPINE1**

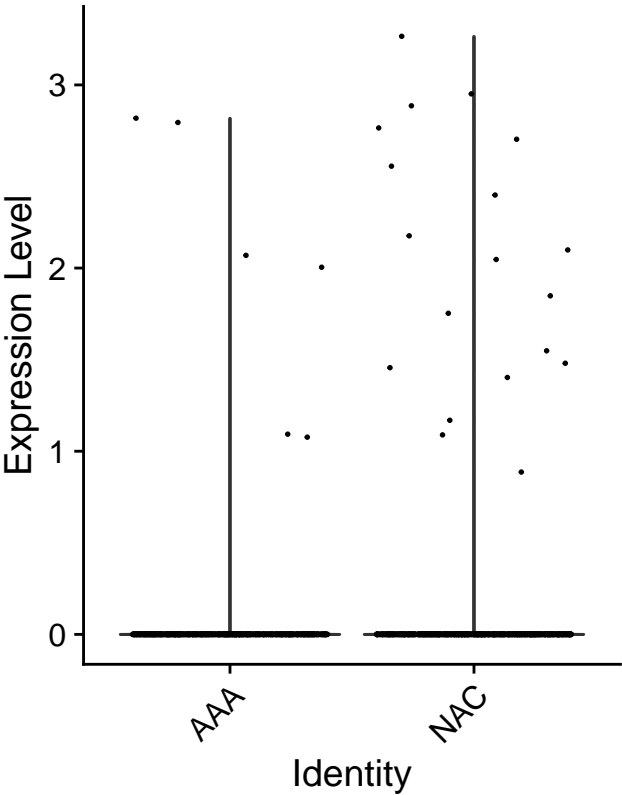

**IL24**

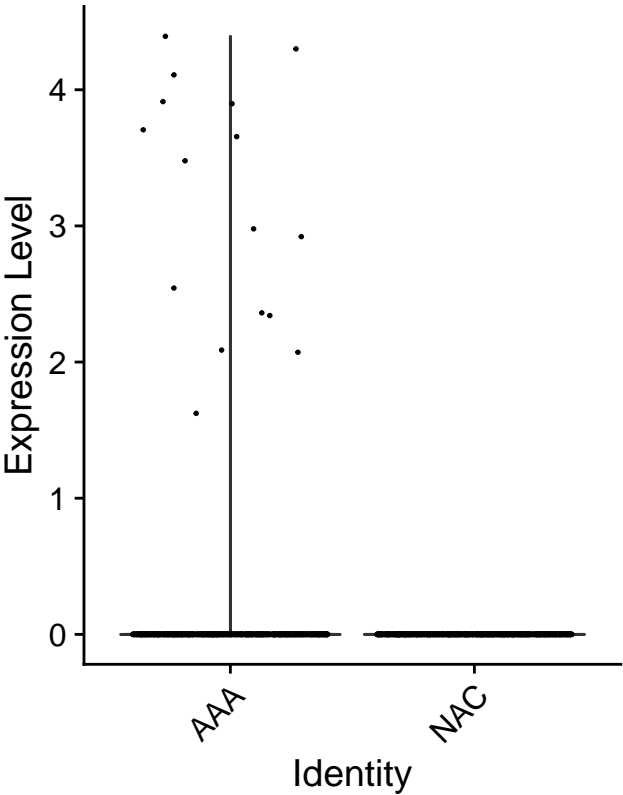

**HMOX1**

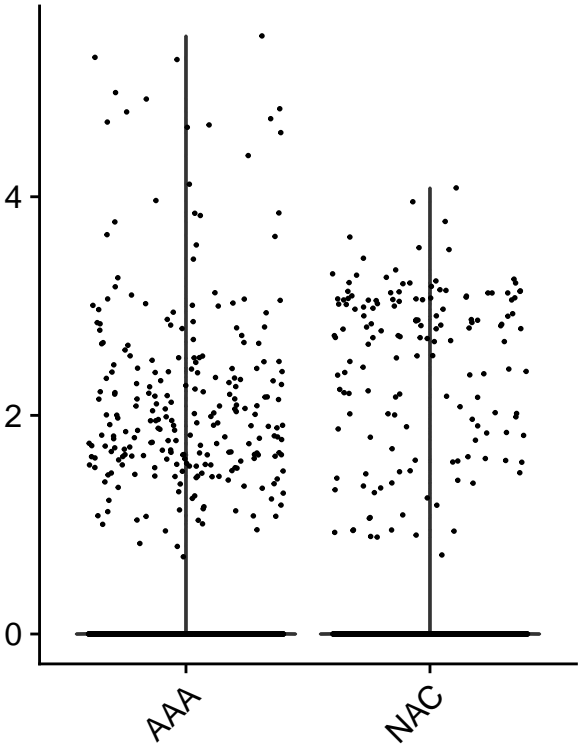

Supplement: Supplementary file 1 [file Data_Sheet_1.ZIP › 8_scRNA_mono/ann_marker_VlnPlot.pdf]

**AAA**

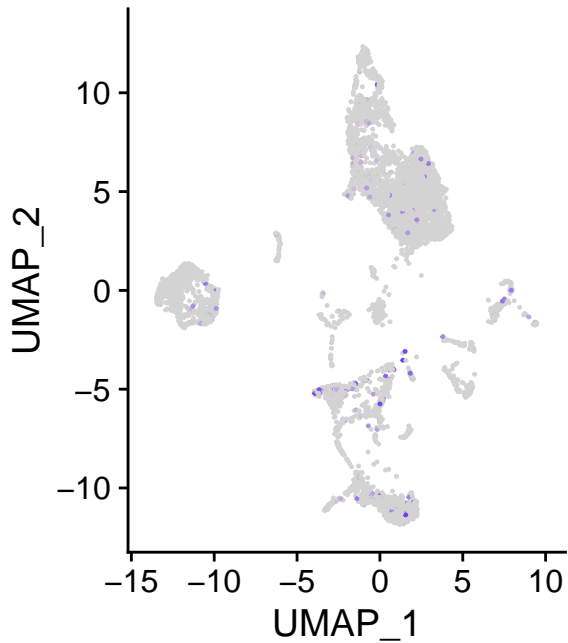

**NAC**

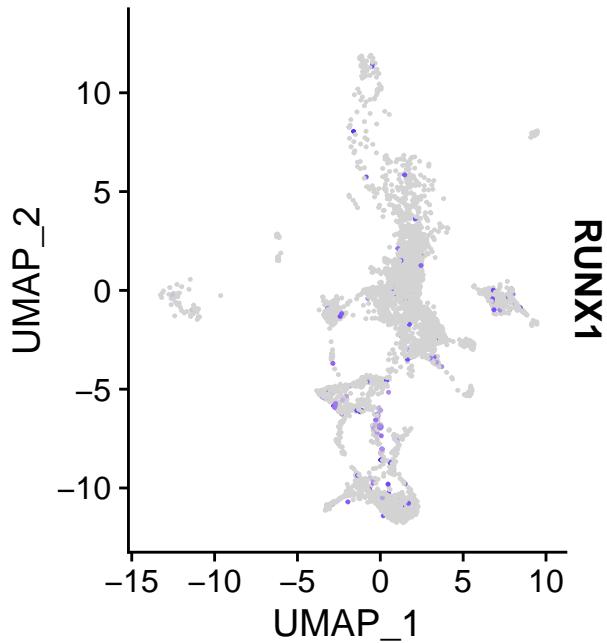

Supplement: Supplementary file 1 [file Data_Sheet_1.ZIP › 8_scRNA_mono/gene_FeaturePlot.pdf]

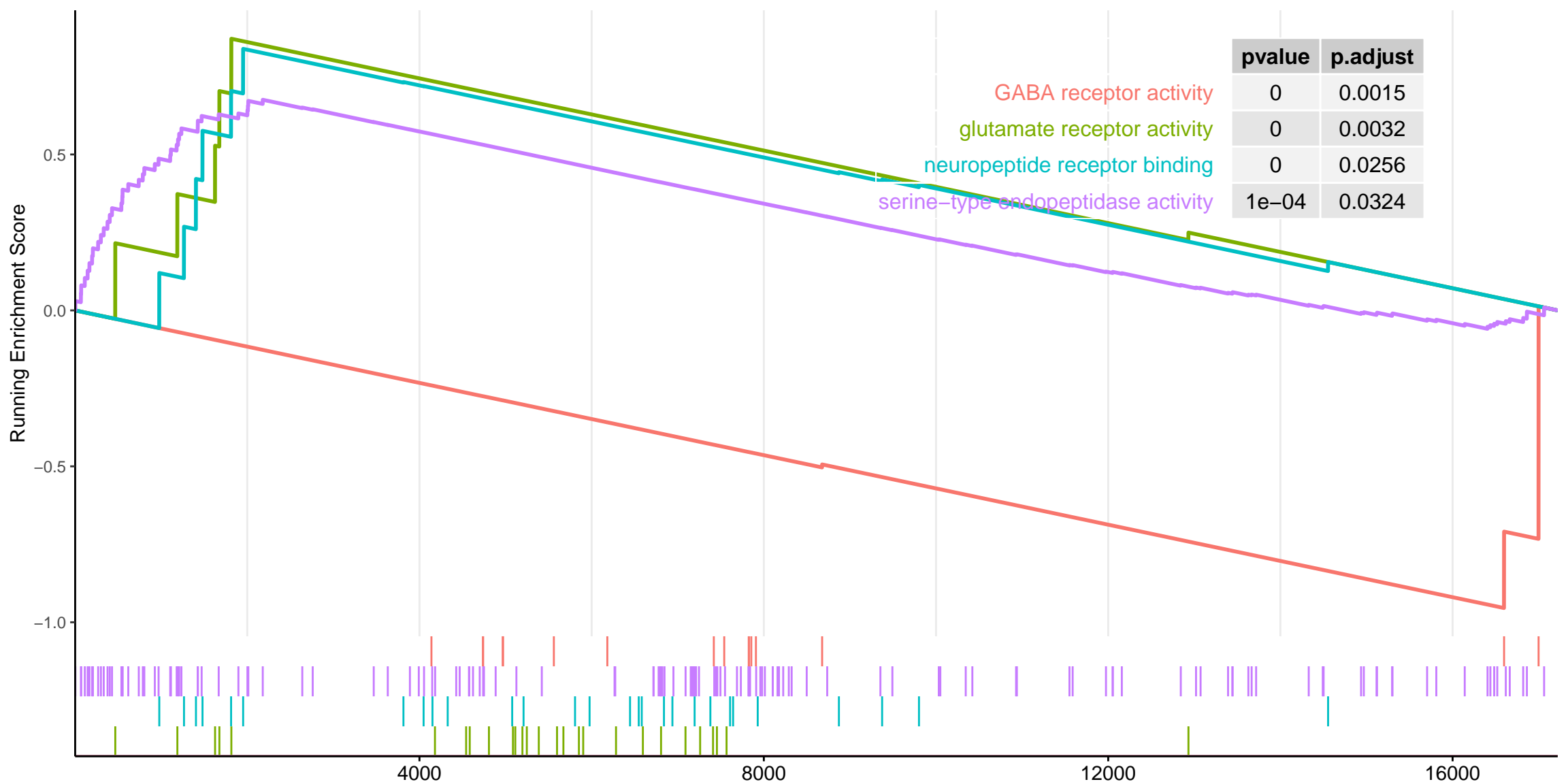

Supplement: Supplementary file 1 [file Data_Sheet_1.ZIP › 8_scRNA_mono/RUNX1_down/RUNX1gseaGO.pdf]

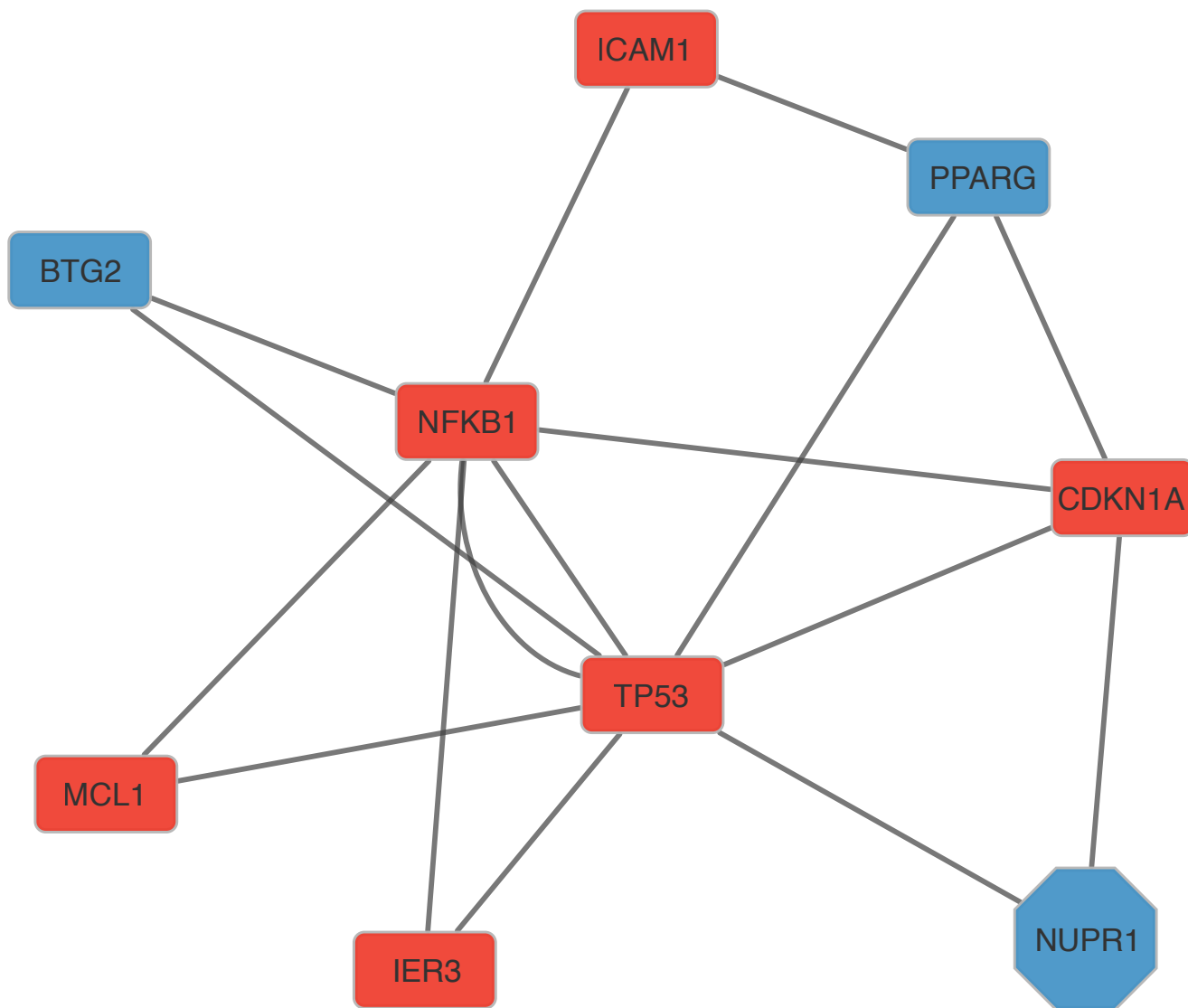

Supplement: Supplementary file 1 [file Data_Sheet_1.ZIP › 9_ppi.mono/1- Cluster 1 (Score- 3.75) copy.pdf]

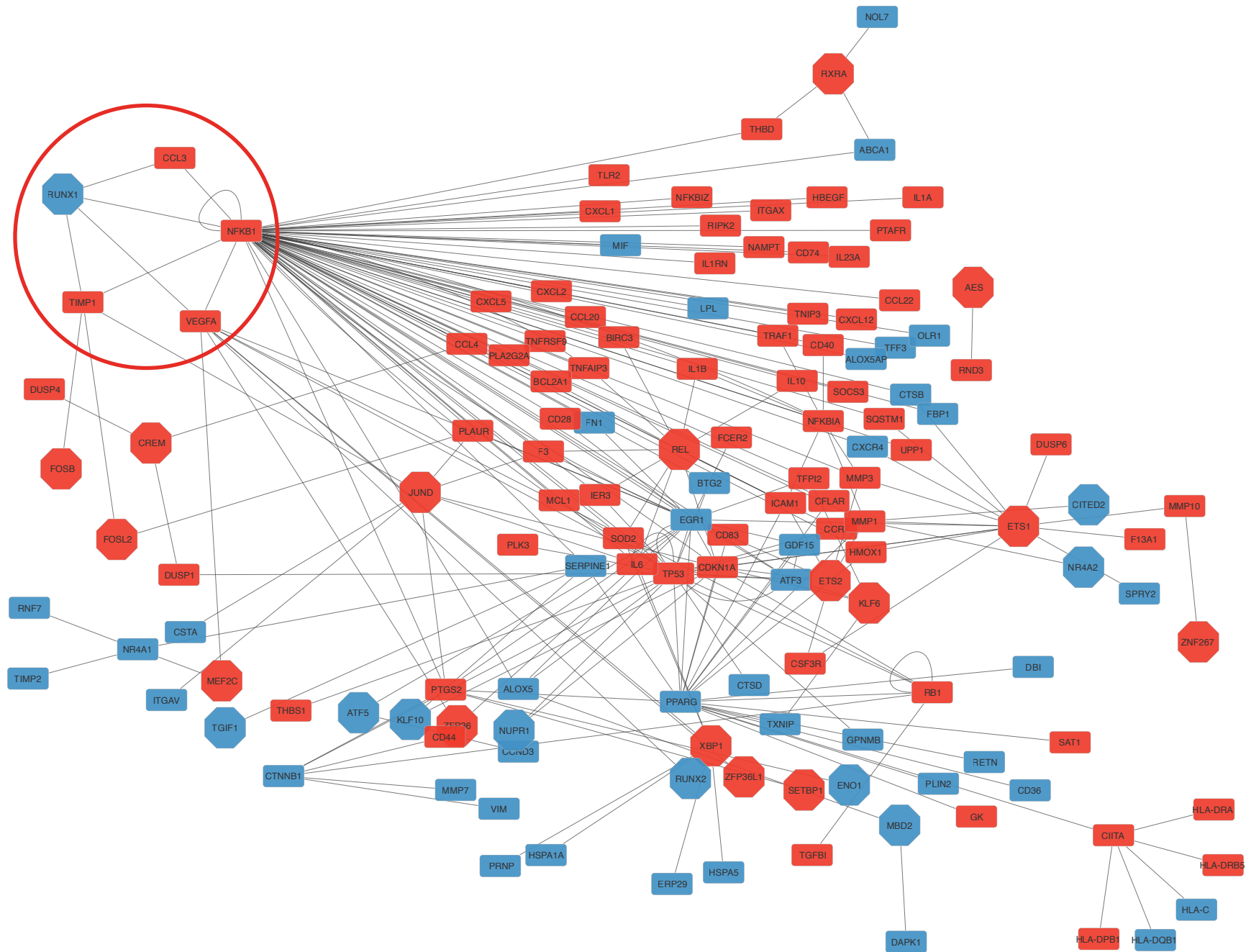

Supplement: Supplementary file 1 [file Data_Sheet_1.ZIP › 9_ppi.mono/tf_mRNA.edge.txt copy.pdf]

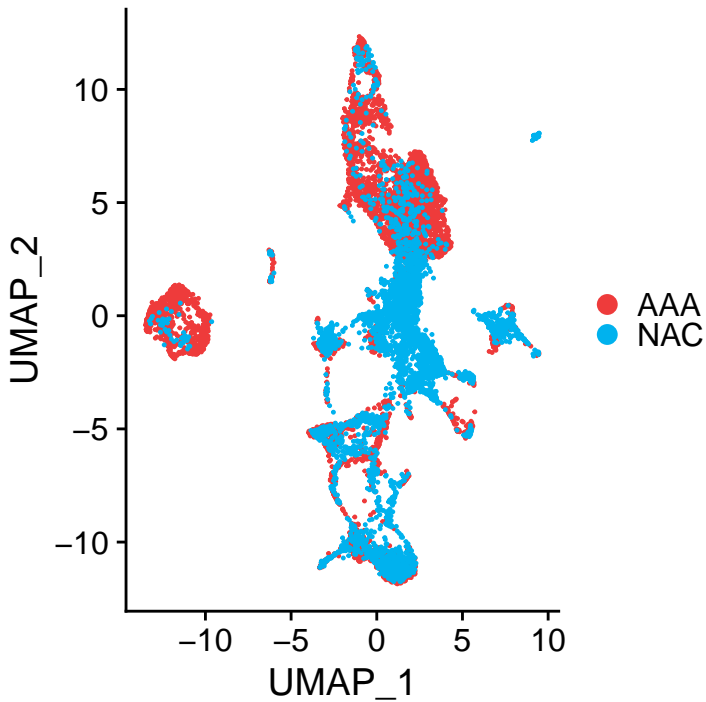

Supplement: Supplementary file 2 [file Data_Sheet_2.ZIP › 3_AAA_singleCell/4.umap/dim_grouptype.pdf]

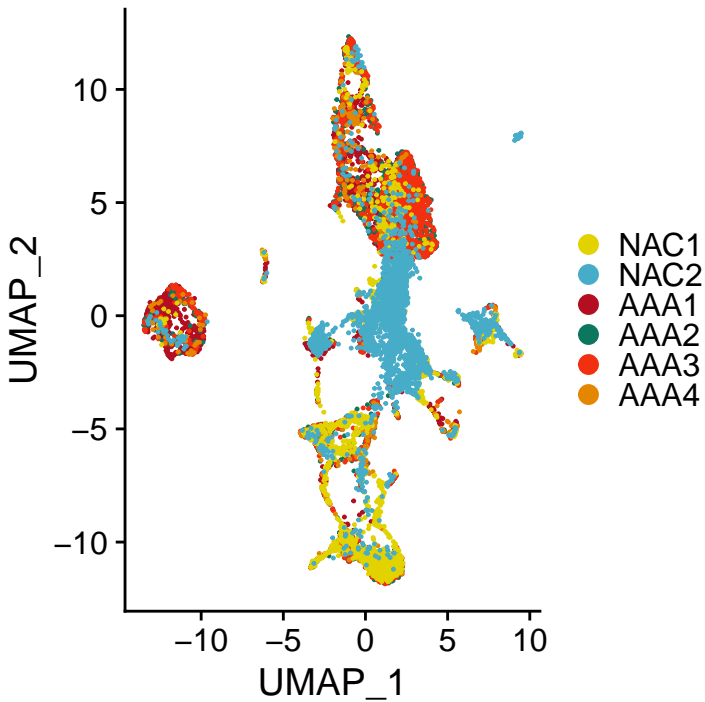

Supplement: Supplementary file 2 [file Data_Sheet_2.ZIP › 3_AAA_singleCell/4.umap/dim_patients.pdf]

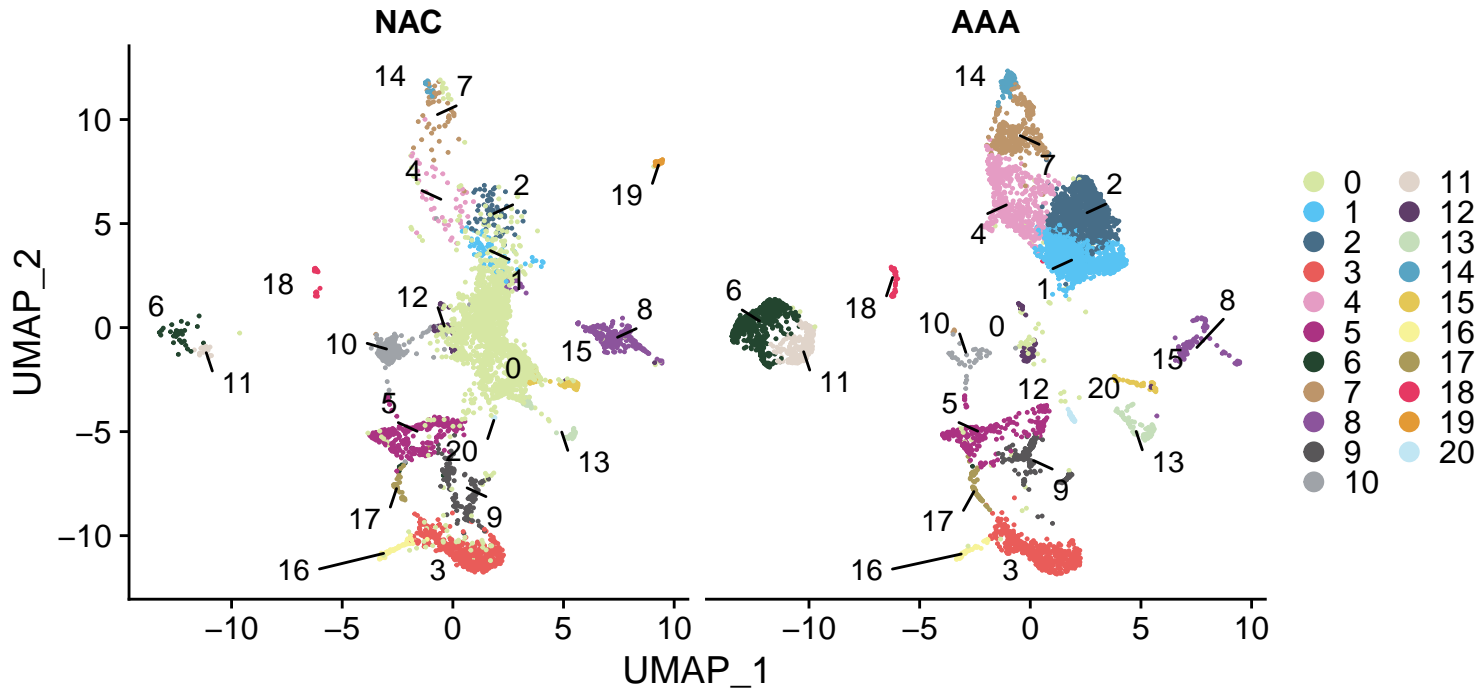

Supplement: Supplementary file 2 [file Data_Sheet_2.ZIP › 3_AAA_singleCell/4.umap/dim_split.pdf]

**celltype\_Monaco**

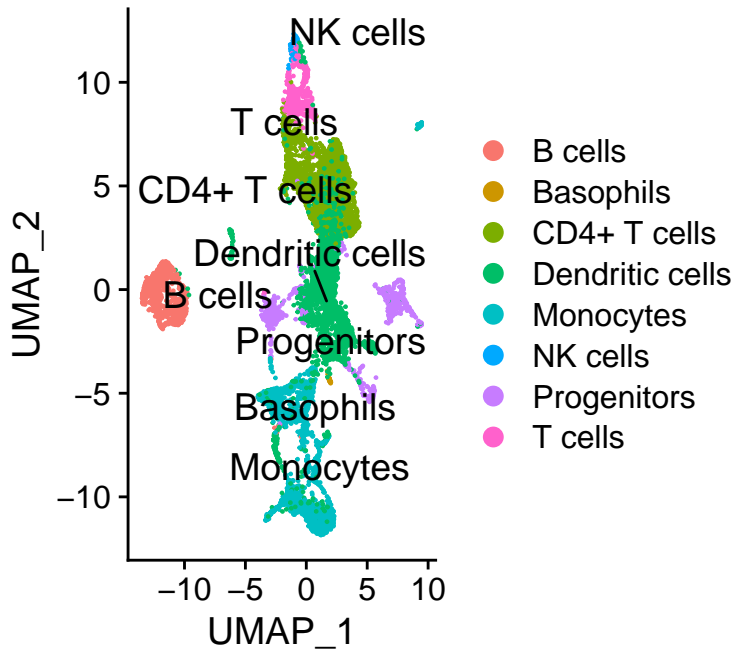

**celltype\_DICE**

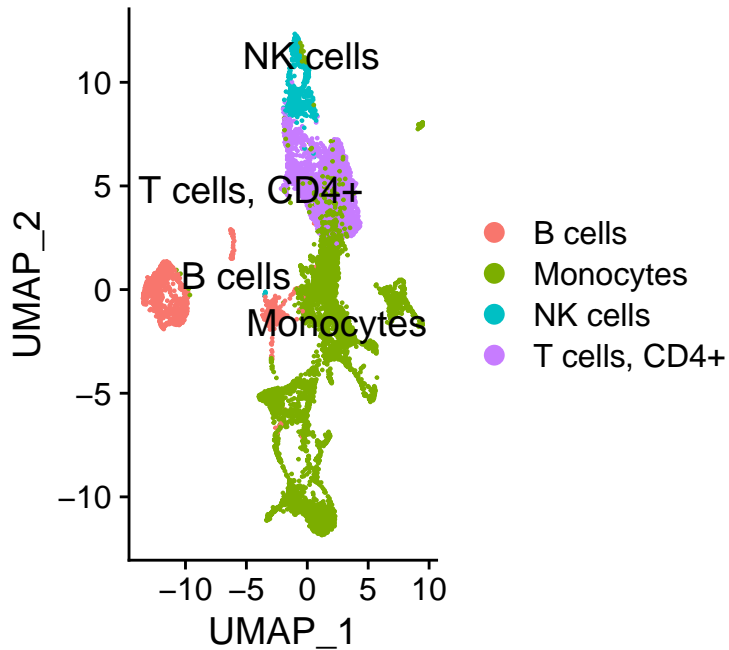

Supplement: Supplementary file 2 [file Data_Sheet_2.ZIP › 3_AAA_singleCell/5.1.ann_singler/Monaco_DICE.pdf]

# celltype\_DICE

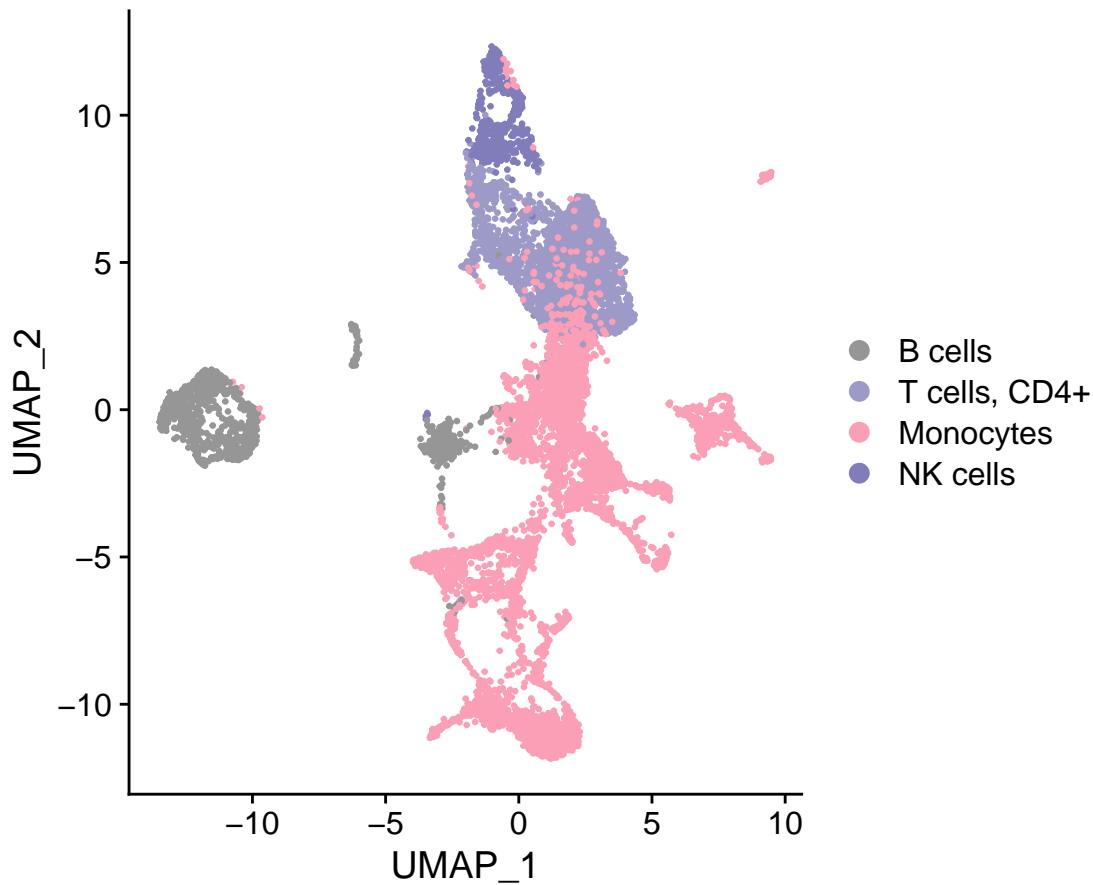

Supplement: Supplementary file 2 [file Data_Sheet_2.ZIP › 3_AAA_singleCell/5.1.ann_singler/UMAP_celltype_DICE.pdf]

# celltype\_Monaco

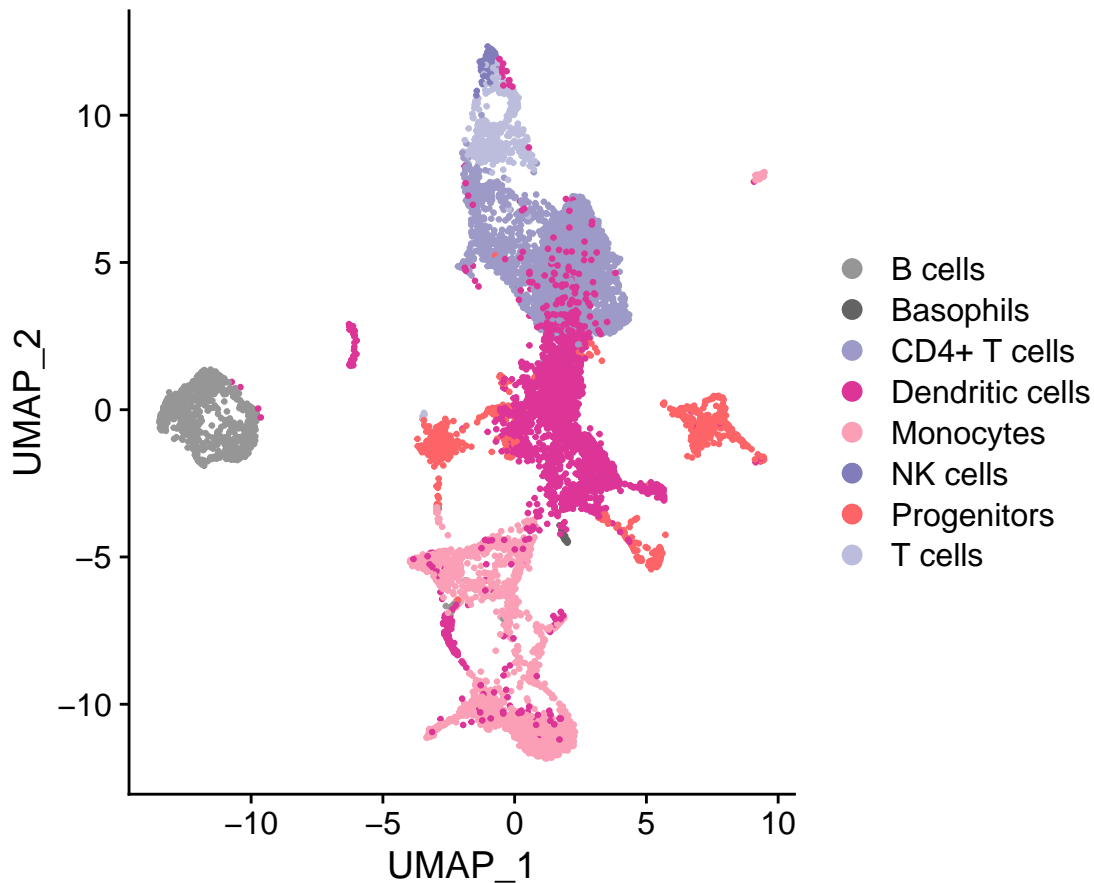

Supplement: Supplementary file 2 [file Data_Sheet_2.ZIP › 3_AAA_singleCell/5.1.ann_singler/UMAP_celltype_Monaco.pdf]

**CSF1R**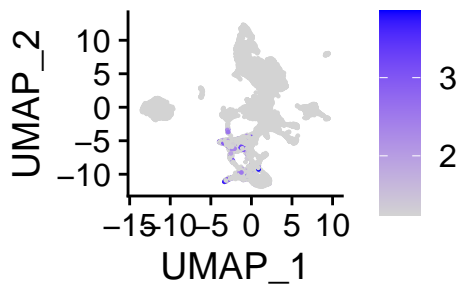**CD14**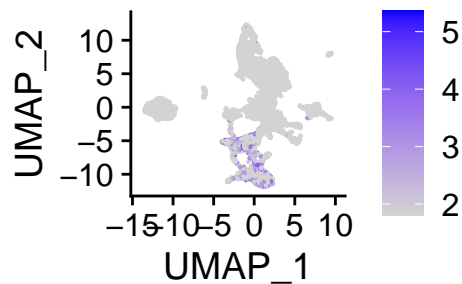**CD68**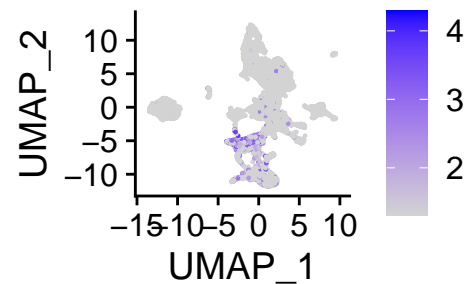**CD2**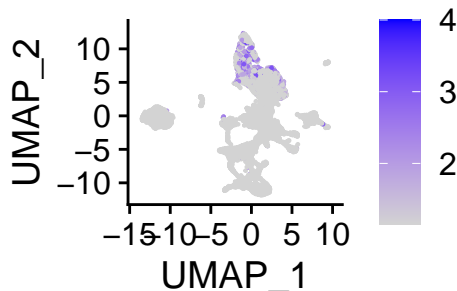**CD3D**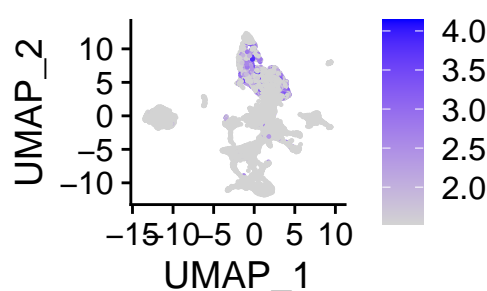**PRF1**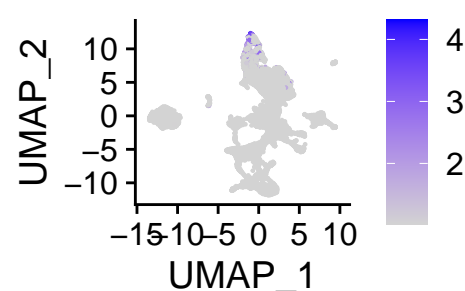**KLRF1**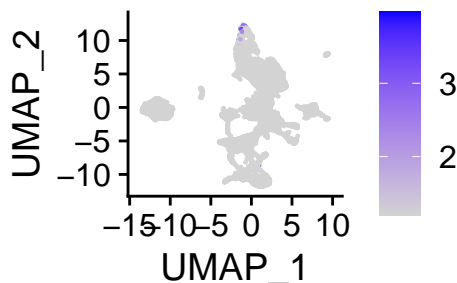**ITGAX**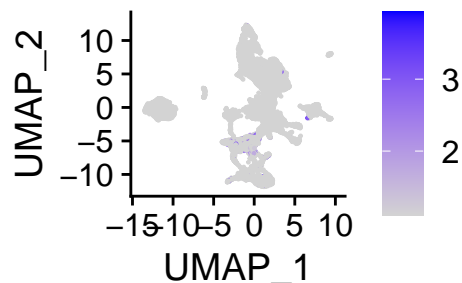**CD19**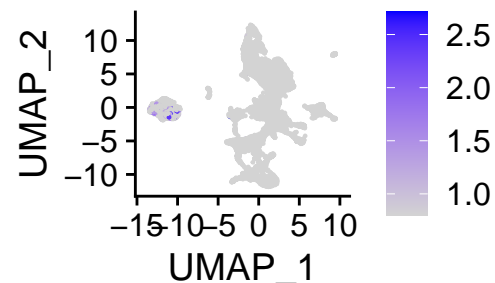

Supplement: Supplementary file 2 [file Data_Sheet_2.ZIP › 3_AAA_singleCell/5.2.ann_marker/5.2.ann_marker_FeaturePlot.pdf]

**CSF1R**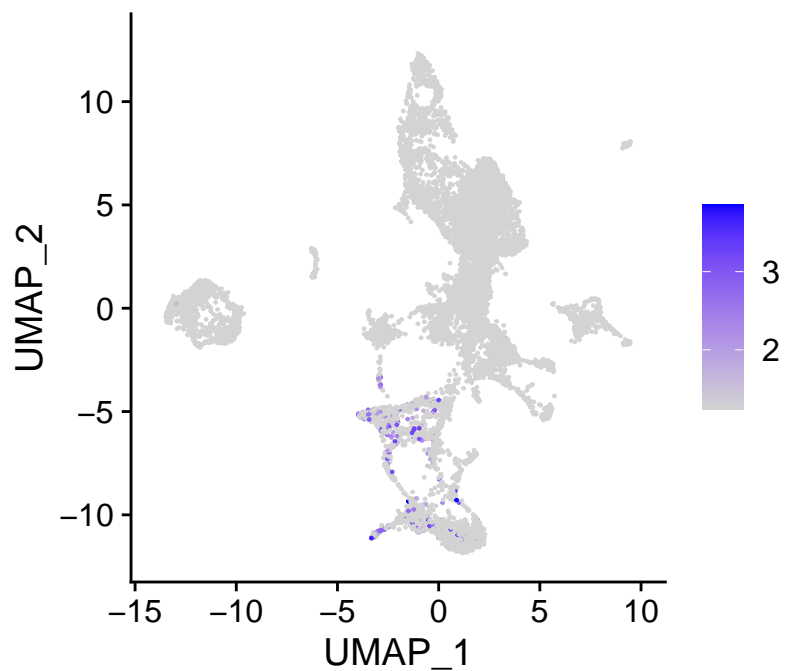**CD14**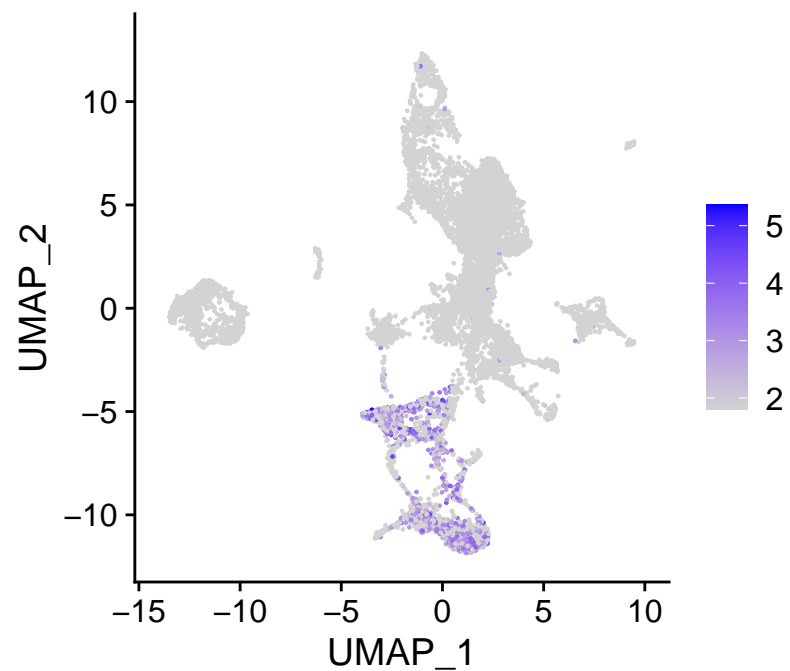**CD68**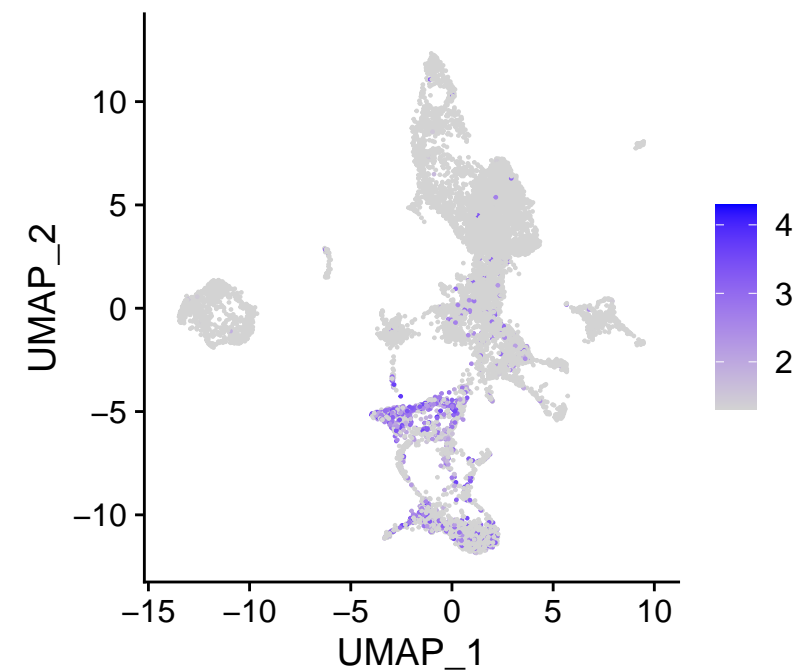**CD2**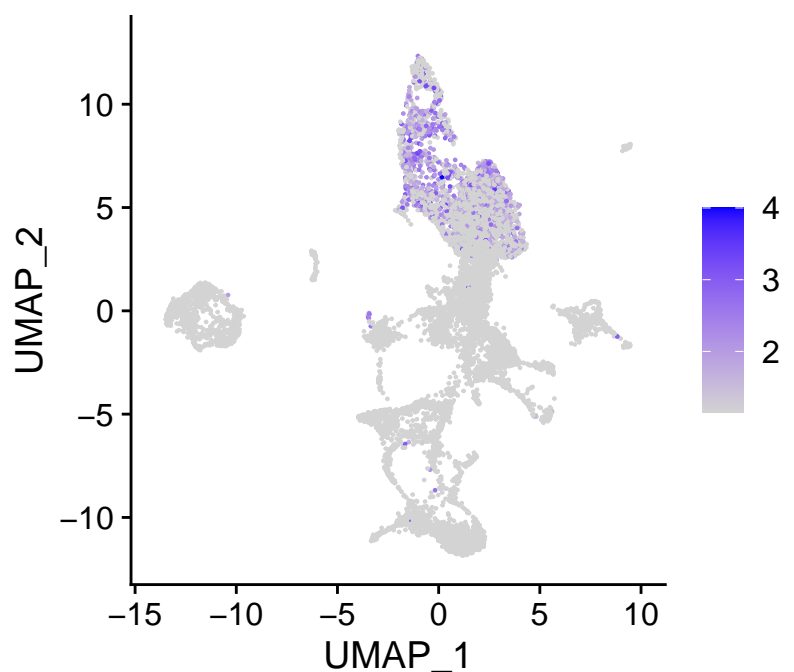**CD3D**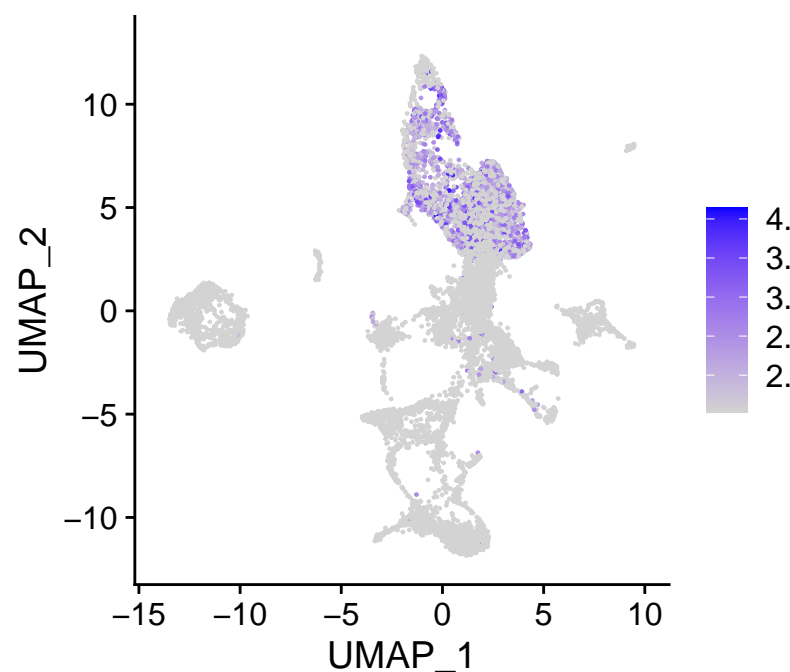**PRF1**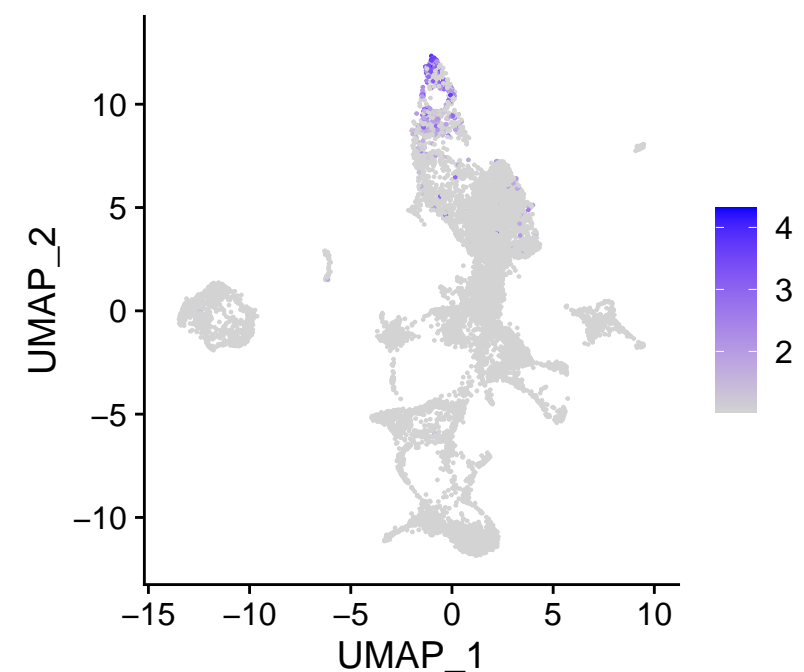**KLRF1**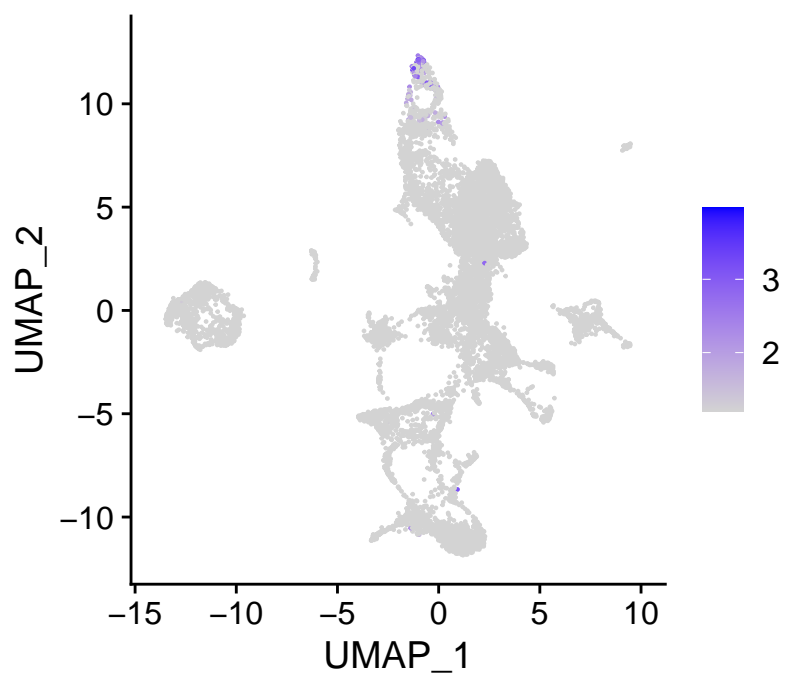**ITGAX**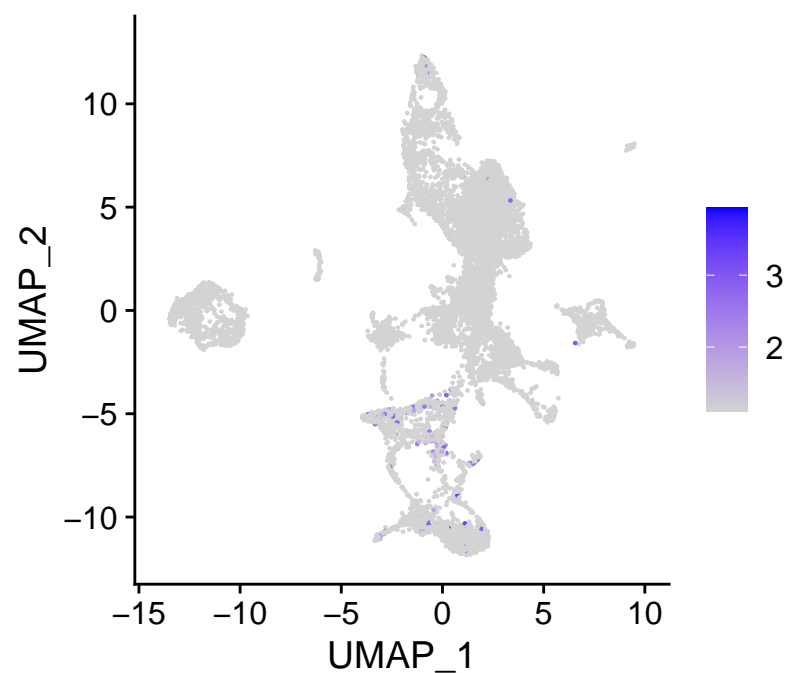**CD19**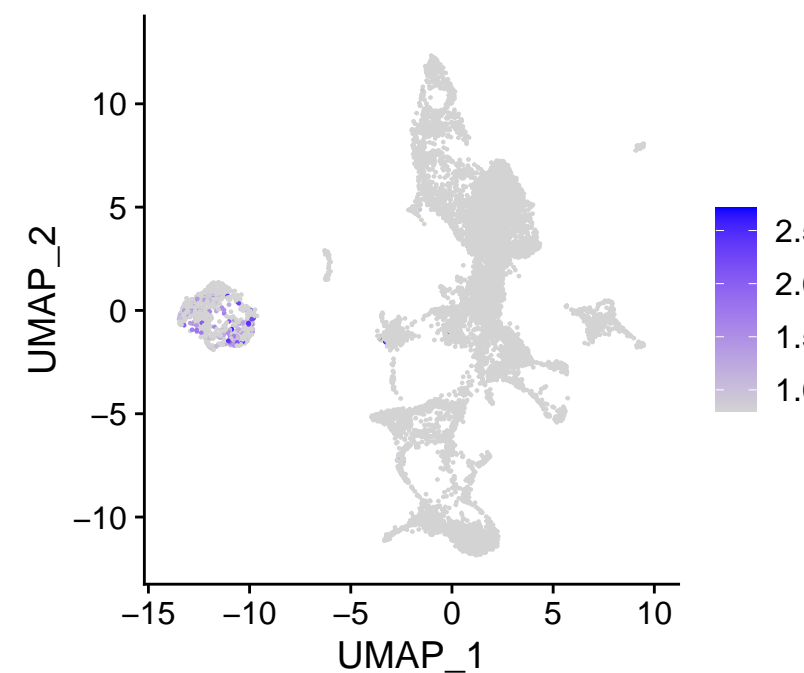

Supplement: Supplementary file 2 [file Data_Sheet_2.ZIP › 3_AAA_singleCell/5.2.ann_marker/ann_marker_FeaturePlot.pdf]

Cell clusters

0  
1  
2  
3  
4  
5  
6  
7  
8  
9  
10  
11  
12  
13  
14  
15  
16  
17  
18  
19  
20

CSF1R

CD14

CD68

CD2

CD3D

PRF1

KLRF1

ITGAX

CD19

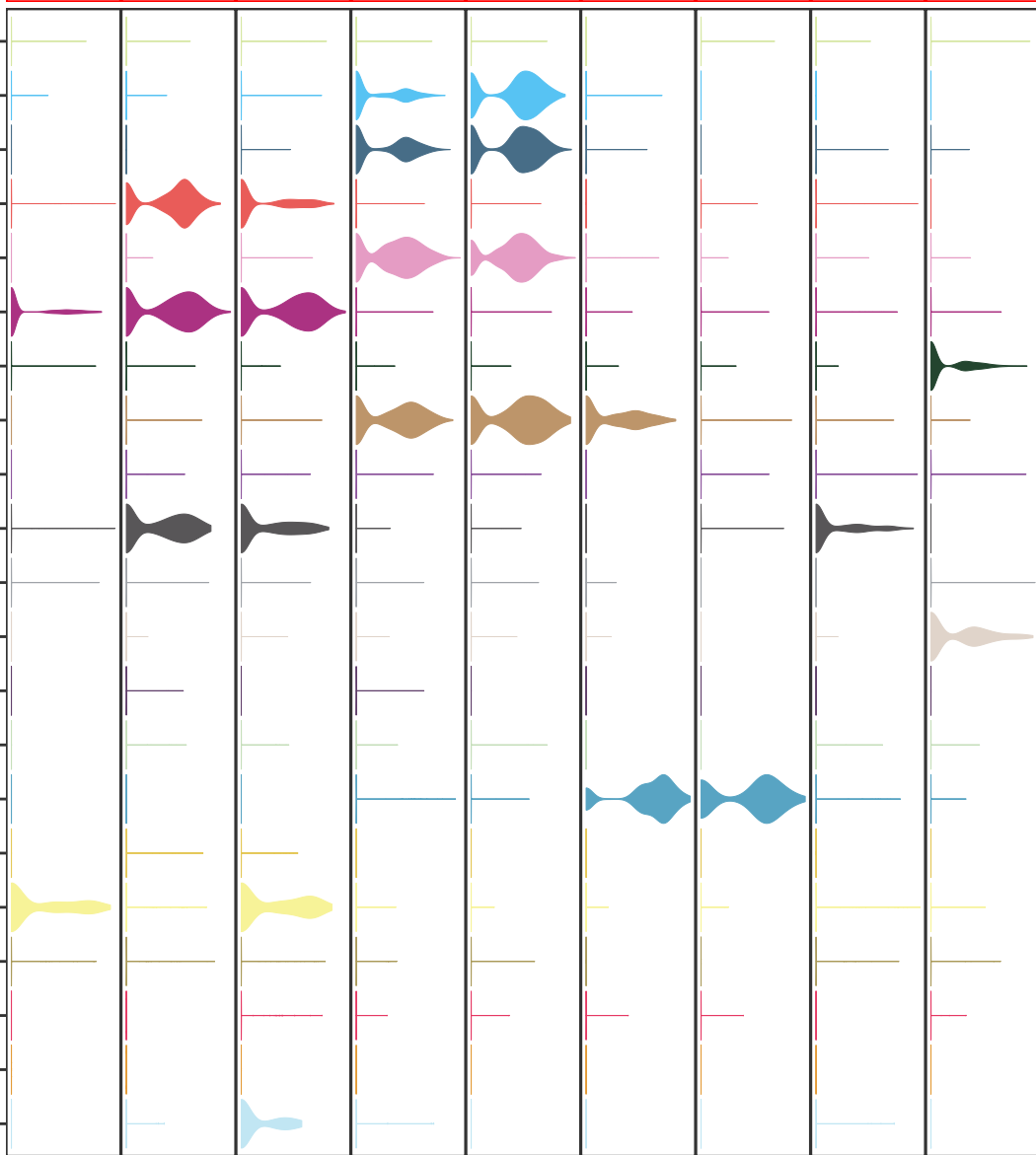

Supplement: Supplementary file 2 [file Data_Sheet_2.ZIP › 3_AAA_singleCell/5.2.ann_marker/ann_marker_VlnPlot.pdf]

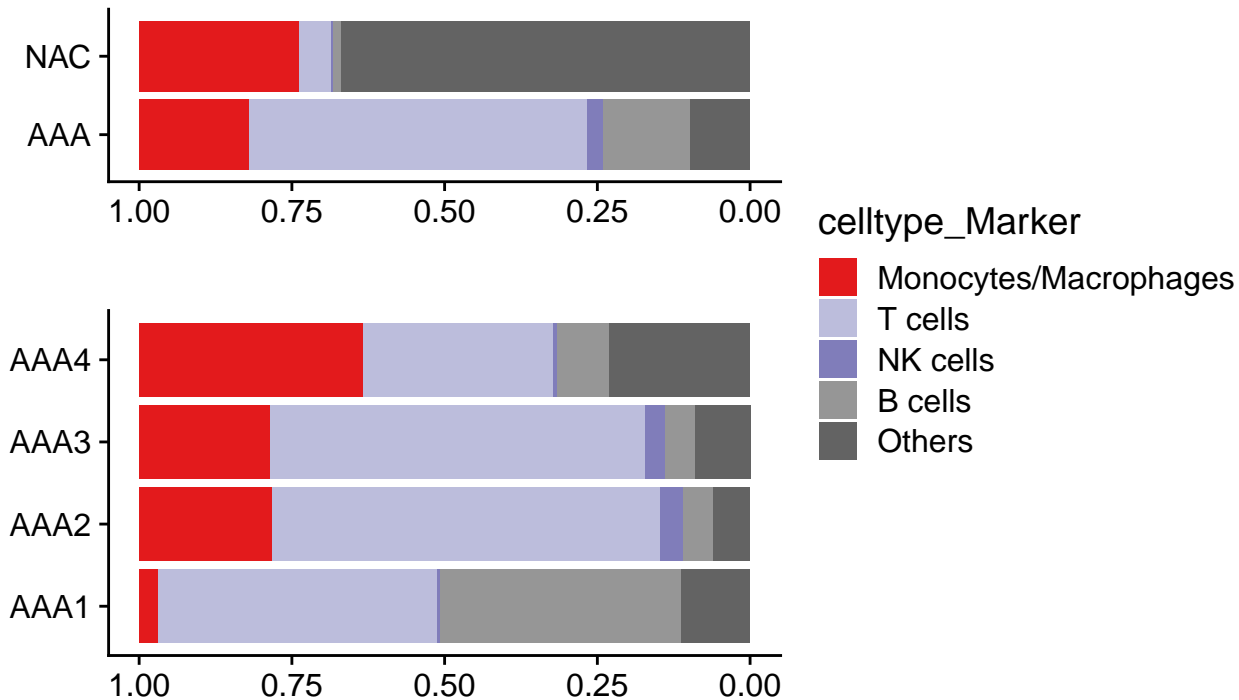

Supplement: Supplementary file 2 [file Data_Sheet_2.ZIP › 3_AAA_singleCell/5.2.ann_marker/barplot_all.pdf]

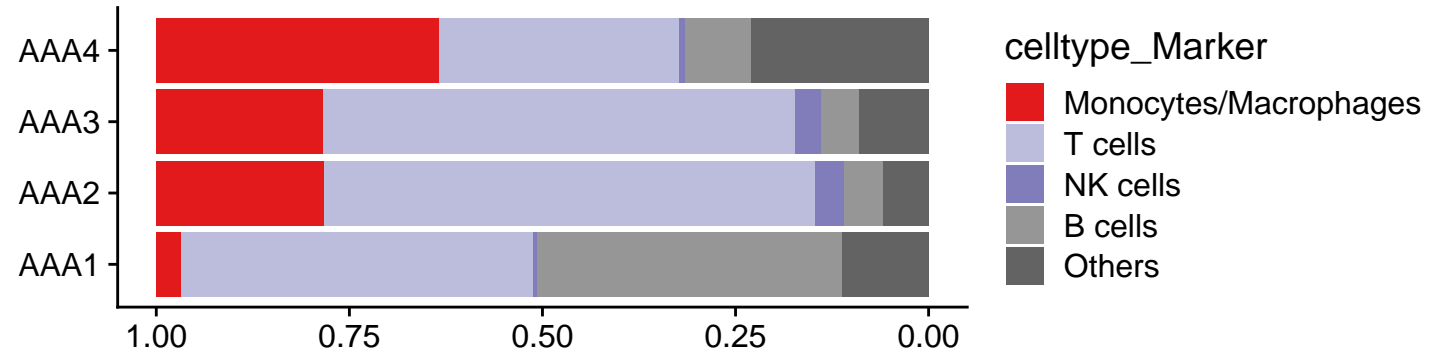

Supplement: Supplementary file 2 [file Data_Sheet_2.ZIP › 3_AAA_singleCell/5.2.ann_marker/barplot_group.pdf]

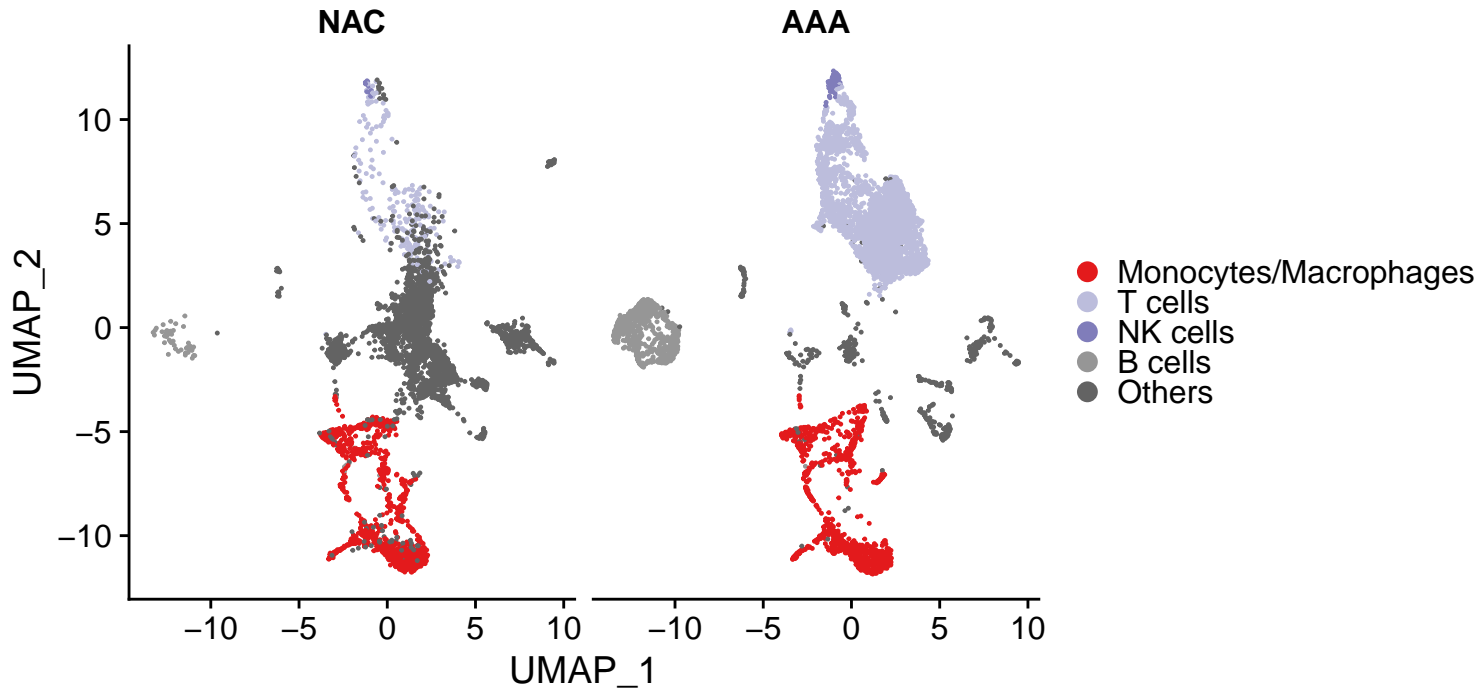

Supplement: Supplementary file 2 [file Data_Sheet_2.ZIP › 3_AAA_singleCell/5.2.ann_marker/cell_ann_umap.pdf]

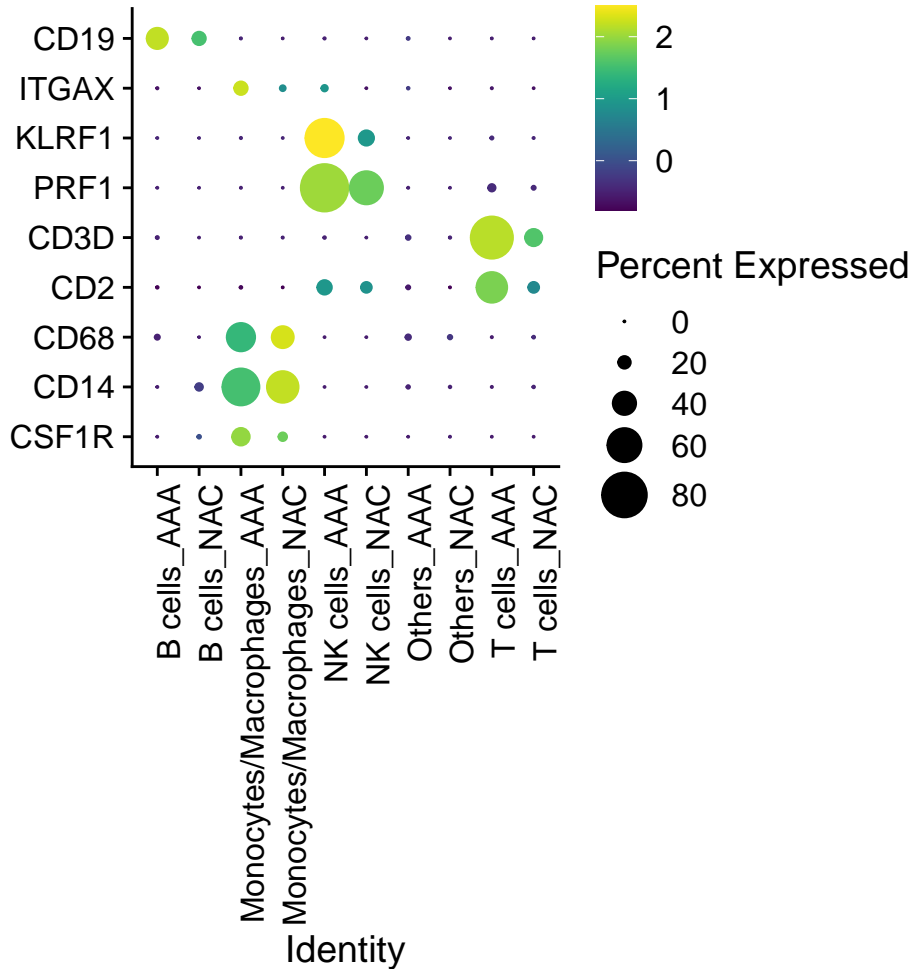

Supplement: Supplementary file 2 [file Data_Sheet_2.ZIP › 3_AAA_singleCell/5.2.ann_marker/cell_marker.pdf]

Color Key

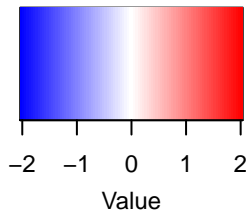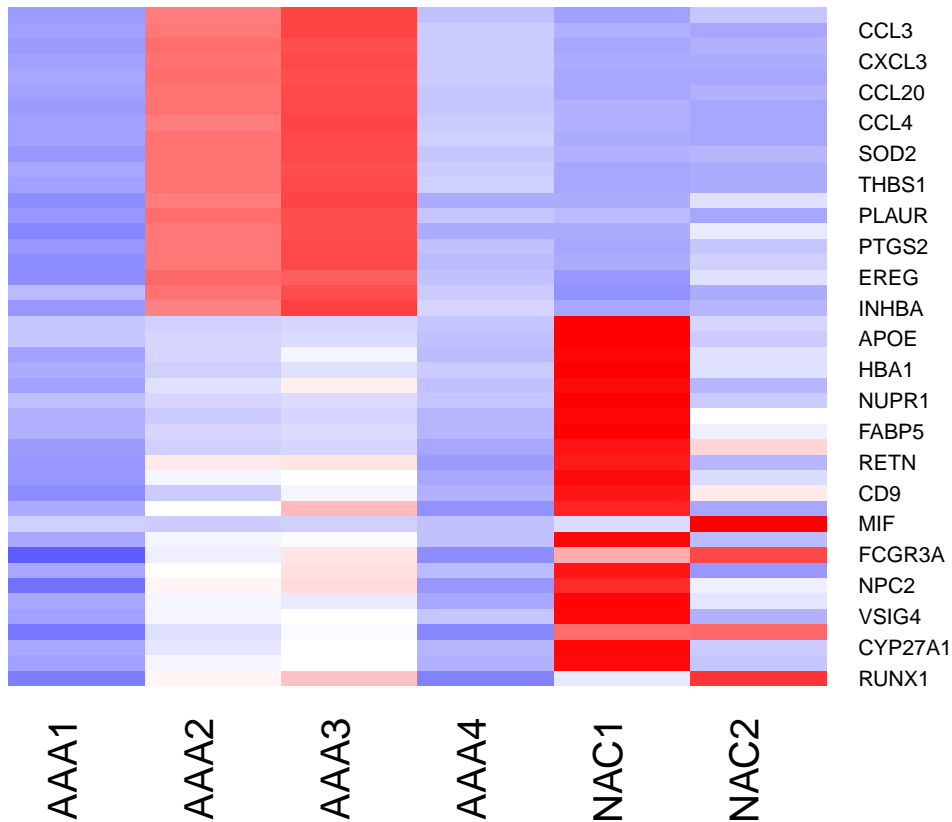

Supplement: Supplementary file 2 [file Data_Sheet_2.ZIP › 3_AAA_singleCell/6.mono.diff/degs.heatmap.pdf]

AAA

NAC

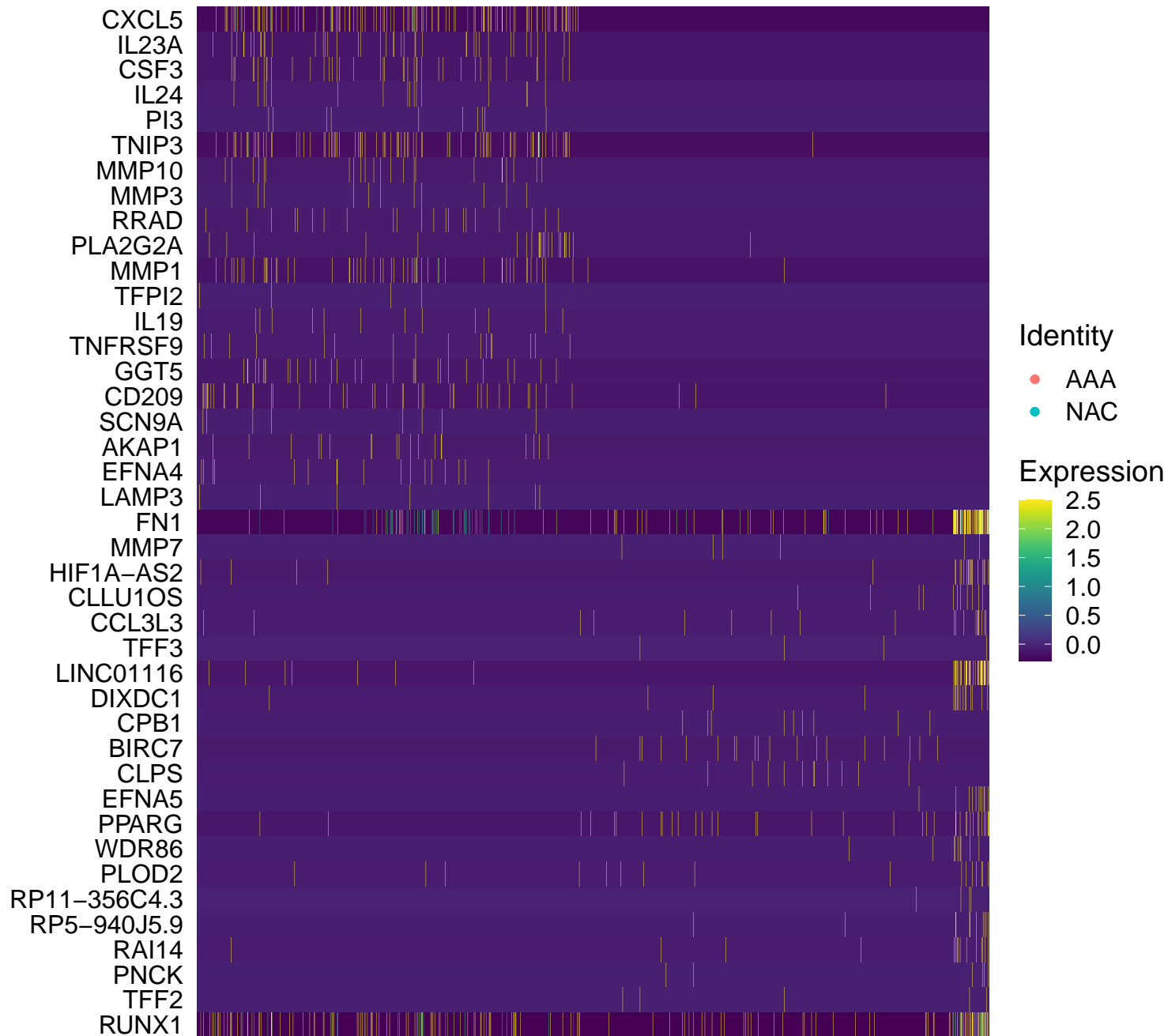

Supplement: Supplementary file 2 [file Data_Sheet_2.ZIP › 3_AAA_singleCell/6.mono.diff/degs_deHeat.pdf]

AAA

NAC

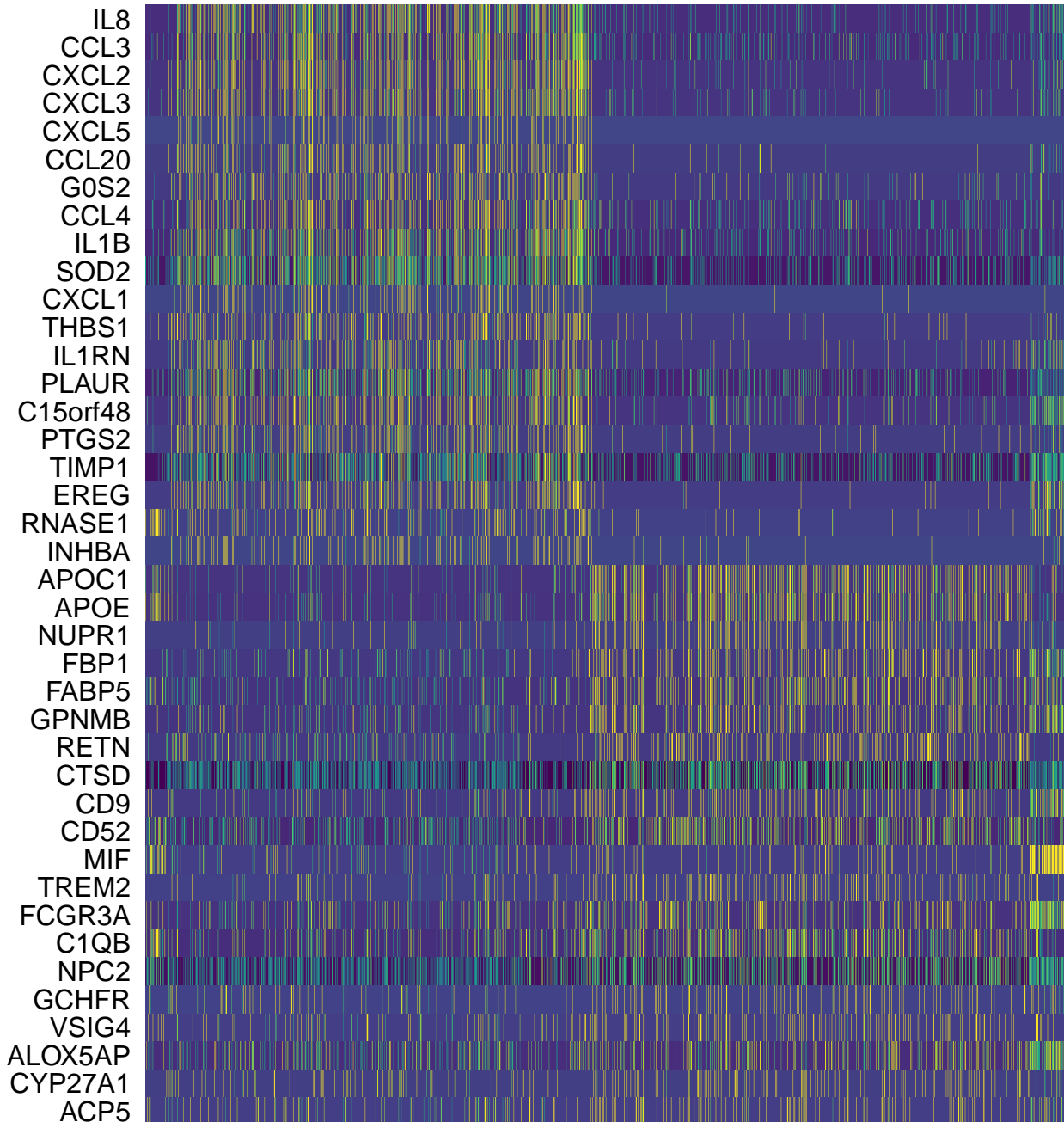

Identity

• AAA

• NAC

Expression

2

1

0

Supplement: Supplementary file 2 [file Data_Sheet_2.ZIP › 3_AAA_singleCell/6.mono.diff/mono_deHeat.pdf]
