# Supplementary material for: Intracranial Aneurysms Induced by RUNX1 Through Regulation of NFKB1 in Patients With Hypertension-An Integrated Analysis Based on Multiple Datasets and Algorithms
Source: Front Neurol. 2022 May 17;13:877801. doi: 10.3389/fneur.2022.877801 (PMC9152011; doi:10.3389/fneur.2022.877801)
Supplement: Supplementary file 1 [file Data_Sheet_1.ZIP › 8_scRNA_mono/RUNX1_up/plot/GSEA可视化-分析报告_2022-01-09_23_45_46.zip!/output/GSEA可视化.pdf]

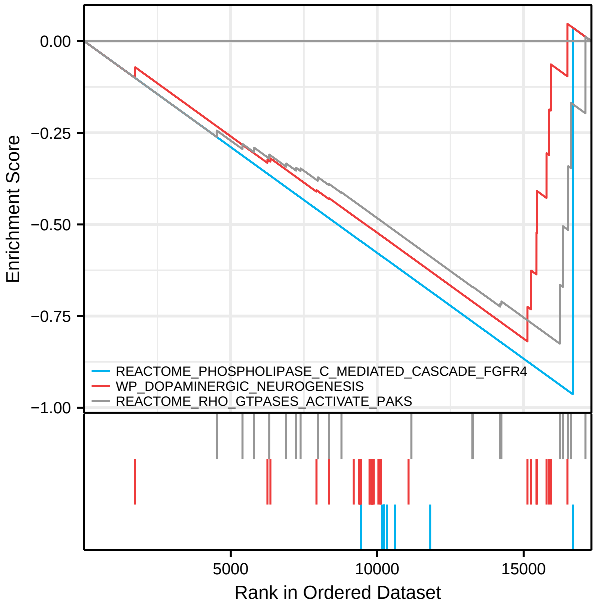

0.00
−0.25
−0.50
−0.75
−1.00
Enrichment Score
REACTOME_PHOSPHOLIPASE_C_MEDIATED_CASCADE_FGFR4
WP_DOPAMINERGIC_NEUROGENESIS
REACTOME_RHO_GTPASES_ACTIVATE_PAKS
5000
10000
15000
Rank in Ordered Dataset
