# Supplementary material for: Intracranial Aneurysms Induced by RUNX1 Through Regulation of NFKB1 in Patients With Hypertension-An Integrated Analysis Based on Multiple Datasets and Algorithms
Source: Front Neurol. 2022 May 17;13:877801. doi: 10.3389/fneur.2022.877801 (PMC9152011; doi:10.3389/fneur.2022.877801)
Supplement: Supplementary file 1 [file Data_Sheet_1.ZIP › 8_scRNA_mono/RUNX1_up/plot/GSEA可视化-分析报告_2022-01-09_23_45_46.zip!/GSEA可视化-分析报告.html]

仙桃学术报告


GSEA可视化
报告

报告生成时间：
2022-01-09 23:45:46

- 主要结果
- 分析情况

- \* 所选GSEA基因集情况表

- 方法学

◇ 主要结果

  

GSEA可视化

PDF下载

◇ 结果情况

  

所选GSEA基因集情况表

ID

ES

NES

p.adjust

FDR

REACTOME\_PHOSPHOL...

-0.963

-1.678

0.001

1.000

WP\_DOPAMINERGIC\_N...

-0.819

-1.609

0.002

1.000

REACTOME\_RHO\_GTPA...

-0.825

-1.547

0.004

1.000

GSEA基因集情况：

在REACTOME\_PHOSPHOLIPASE\_C\_MEDIATED\_CASCADE\_FGFR4基因集富集不显著（NES = -1.678; p.adjust = 0.001; FDR = 1.000）；

在WP\_DOPAMINERGIC\_NEUROGENESIS基因集富集不显著（NES = -1.609; p.adjust = 0.002; FDR = 1.000）；

在REACTOME\_RHO\_GTPASES\_ACTIVATE\_PAKS基因集富集不显著（NES = -1.547; p.adjust = 0.004; FDR = 1.000）。

· 备注：一般认为显著富集的阈值为：False discovery rate (FDR)<0.25且p.adjust<0.05

  

◇ 方法学

  

· 软件：R (3.6.3版本) (统计分析与可视化)

· R包：主要为 ggplot2包[3.3.3版本] (用于可视化)

· 基因集ID：
REACTOME\_PHOSPHOLIPASE\_C\_MEDIATED\_CASCADE\_FGFR4; WP\_DOPAMINERGIC\_NEUROGENESIS; REACTOME\_RHO\_GTPASES\_ACTIVATE\_PAKS
